# From Motor-Output to Connectivity: An In-Depth Study of *in-vitro* Rhythmic Patterns in the Cockroach *Periplaneta americana*

**DOI:** 10.3389/finsc.2021.655933

**Published:** 2021-05-20

**Authors:** Izhak David, Amir Ayali

**Affiliations:** ^1^School of Zoology, Tel Aviv University, Tel Aviv, Israel; ^2^Sagol School of Neuroscience, Tel Aviv University, Tel Aviv, Israel

**Keywords:** locomotion control, central pattern generator, cockroach, levator, depressor, pilocarpine, intersegmental coordination, coupling strength

## Abstract

The cockroach is an established model in the study of locomotion control. While previous work has offered important insights into the interplay among brain commands, thoracic central pattern generators, and the sensory feedback that shapes their motor output, there remains a need for a detailed description of the central pattern generators' motor output and their underlying connectivity scheme. To this end, we monitored pilocarpine-induced activity of levator and depressor motoneurons in two types of novel *in-vitro* cockroach preparations: isolated thoracic ganglia and a whole-chain preparation comprising the thoracic ganglia and the subesophageal ganglion. Our data analyses focused on the motoneuron firing patterns and the coordination among motoneuron types in the network. The burstiness and rhythmicity of the motoneurons were monitored, and phase relations, coherence, coupling strength, and frequency-dependent variability were analyzed. These parameters were all measured and compared among network units both within each preparation and among the preparations. Here, we report differences among the isolated ganglia, including asymmetries in phase and coupling strength, which indicate that they are wired to serve different functions. We also describe the intrinsic default gait and a frequency-dependent coordination. The depressor motoneurons showed mostly similar characteristics throughout the network regardless of interganglia connectivity; whereas the characteristics of the levator motoneurons activity were mostly ganglion-dependent, and influenced by the presence of interganglia connectivity. Asymmetries were also found between the anterior and posterior homolog parts of the thoracic network, as well as between ascending and descending connections. Our analyses further discover a frequency-dependent inversion of the interganglia coordination from alternations between ipsilateral homolog oscillators to simultaneous activity. We present a detailed scheme of the network couplings, formulate coupling rules, and review a previously suggested model of connectivity in light of our new findings. Our data support the notion that the inter-hemiganglia coordination derives from the levator networks and their coupling with local depressor interneurons. Our findings also support a dominant role of the metathoracic ganglion and its ascending output in governing the anterior ganglia motor output during locomotion in the behaving animal.

## Introduction

Insect hexapedal design is known to enable very stable and highly adaptable locomotion (1–4). These abilities intrigue both neuroethologists, who study the mechanisms underlying animal behavior, and researchers of bioinspired locomotion systems and their controllers (5–10). Functional coordination is achieved, in all legged locomotion, through a dynamic interplay between brain descending commands (11, 12), local central pattern generator networks [CPGs; see reviews (13–15)], and sensory feedback, which modify and adapt the endogenous motor-pattern to suit the behavioral context and environment (16–20). The convention states that slow-walking insects, or animals navigating through a complex environment, mostly depend on sensory feedback and weak central coupling to coordinate their limbs; while fast-walking insects relay more on strong central coupling and a feedforward control strategy (21). We note that feedback and feedforward control refer to the extent to which the endogenous oscillators' (e.g., CPGs) frequencies are influenced by those of the corresponding actuators, as manifests in the proprioceptors' afferents (21). In addition, central and local control refer to the extent to which an hemiganglionic oscillator's activity is influenced by that of its neighbors. Central control is mediated via central connectivity between hemiganglionic networks, while local control is governed by sensory feedback from the hemiganglionic proprioceptors, as well as sensory feedback mediated inputs from other sensors. Although all insects share the same basic architecture of their central nervous system (22), the behavior it generates varies greatly within and between species. Among the leading insect models for locomotion control research, the slow-walking stick insect and the remarkably fast American cockroach (*Periplaneta americana*) present two extreme examples of these control strategies (23), while a third common model, the locust, fits somewhere in-between (24). Insects usually demonstrate one of three prototypical inter-leg coordination patterns or gaits: metachronal wave; tetrapod; or double-tripod (hereafter tripod), in which five, four, or three legs, respectively, are simultaneously maintained on the ground at any given time (25–27). Intermediate footfall patterns that cannot be classified as one of the prototypical gaits have been reported [cockroach (25, 27); *Drosophila* (28–30); stick insect (31, 32)]. Insects alter their gait either in response to changing circumstances (33, 34), or to adapt leg-coordination in response to a change in as little as a single speed-related parameter, like a load sensor (35, 36); as also seen in the speed-dependent phase-shift toward ideal tripod phases in intact and semi-intact deafferented cockroaches (37, 38). Most insects increase their speed by increasing stride frequency up to a certain speed, and then increase stride length to reach their maximum speed (39). *P. americana* is unique in that it can increase both stride frequency by 30% and stride length by up to 300%, due to its extremely long hind legs and extraordinary ability to fast cycle them, which enables it to reach a top speed of 1.5 m/s, or 50 body length per second (1, 37). During fast locomotion the hind legs extend farther to increase stride length and cover greater distance, while hardly changing the duration of the swing (the leg's airborne phase), by increasing swing velocity, as also found in flies (40, 41). The insect leg incorporates three main leg-joints: the thorax-coxa, the coxa-trochanter, and the femur-tibia. Studies of pilocarpine-stimulated preparations suggests that each joint is controlled by a dedicated CPG (42, 43), which also maintains the coordination with the neighboring joints' CPGs (44). Most research, from the early 1970s on [(45–49); review (23, 43)], focused on the coxa-trochanter joint and its levator-depressor control network, by monitoring the corresponding MNs motor-output. This control network also underlies body propulsion, which is almost exclusively generated by depression torque at the coxa-trochanter joint (50). Recent work on locusts and stick insects focused on the depressor side of the network, following the assumption that the levator mirrors its conjugated depressor activity (51–55). However, this narrative, although useful, is incomplete. Pearson and Iles (1970) observed that in a deafferented cockroach, levator MNs can fire independently of depressor MNs, but never vice versa. This phenomenon was also observed *in-vitro* in locusts (56). In addition, levator MNs, but not depressor MNs, were found to fire in correlation with intersegmental signals recorded from the thoracic connectives of the deafferented cockroach, which led to the suggestion that levator premotor networks are centrally controlled (47). Based on these and other observations, including our own findings [(38) and references within], we have previously suggested a parsimonious connectivity model of the CPGs network in which levator interneurons (INs) are centrally controlled (i.e., directly by the hemisegmental oscillator which shares a common drive with homolog oscillators and is connected to neighboring oscillators by mutual inhibition), while the output of depressor INs is influenced by their neighboring levators and not directly and exclusively by the hemisegmental oscillator (38). In the current study we reexamine our and others' previous findings to fill in major gaps in the architecture of the parsimonious connectivity model and the coupling scheme it is based upon (38). This is crucial for uncovering the details of the central control of insect locomotion and for designing models for CPG-based artificial controllers (57). Here we study in depth the relations between frequency and phase relations, as well as the coupling between the cockroach thoracic CPGs. Throughout, we directly monitored both the depressor and levator nerves in order to study the neural control that underlie the coxa-trochanter joint movements, and to obtain a broader description of the network's intra- and inter-hemisegmental connectivity. We first examined each thoracic ganglion in complete isolation from any sensory, descending, or central intersegmental inputs, in order to identify their intrasegmental connectivity. We then examined a novel whole-chain preparation, comprising the thoracic ganglia connected to the subesophageal ganglion (SEG), in order to investigate the intersegmental connectivity and its effects. The whole-chain preparation was also established in order to enable future research into insect locomotion control using a preparation that generates stable prolonged fictive locomotion rhythms, to which effectors and manipulations can be applied and studied. We therefore included the SEG which is known to generate a drive that sustains activation of the thoracic motor networks and participates in intersegmental (but not intrasegmental) coordination [(11, 52, 53) and ref's within]. Our findings present significant and detailed differences between the thoracic ganglia motor-output, including a first description of bi-phasic frequency-dependent endogenous prothoracic motor-output, differences in the coordination and coupling strength between homolog pairs of MNs, and between the anterior and posterior sub-networks. Our findings of coupling strength are summarized in a comprehensive coupling scheme, and we revisit and update our connectivity model based on our new findings. Finally, this work offers extensive data for a future comprehensive comparative studies of the main insect models used for electrophysiology-based locomotion control research in recent years: the cockroach, the stick insect, and the desert locust.

## Materials and Methods

### Experimental Animals

Experiments were conducted on 22 adult male *Periplaneta americana* cockroaches obtained from our colony at the School of Zoology, Tel-Aviv University. The insects were maintained in a 60-liter plastic cage at a room temperature of 30°C, under a light:dark cycle of 12 h:12 h. Their diet comprised dry cat food (La-Cat, BioPet, Israel) and water *ad libitum*.

### Neurophysiological Procedure

**Figure 1:**
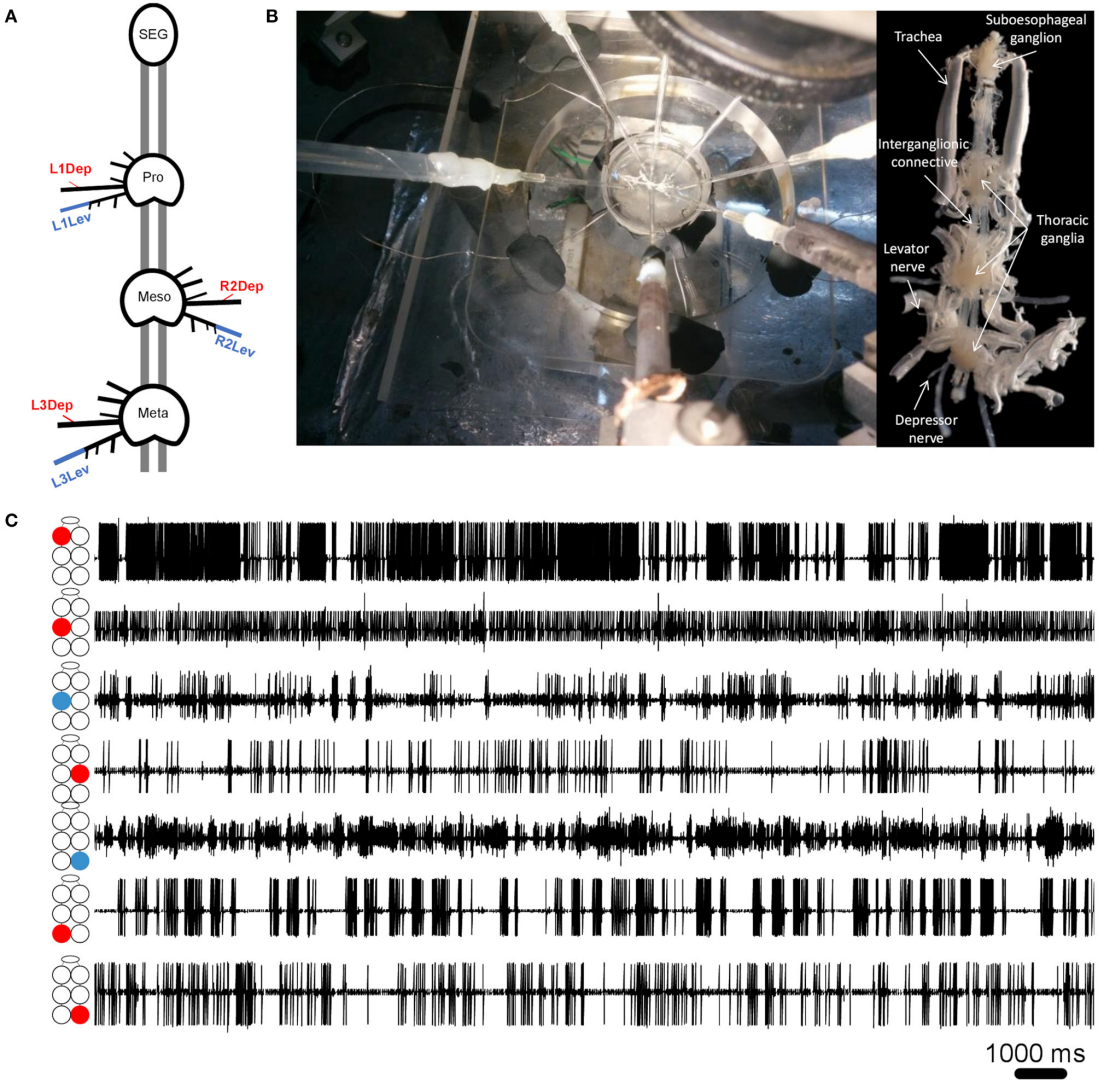
**(A)** Schematic illustration of the whole-chain preparation and the recording sites. Red and blue correspond to depressor and levator nerves, respectively. Nerve nomenclature is presented as side of the body-thoracic ganglion-nerve function. E.g., L1Dep is left-prothoracic-depressor. Vacuum electrodes were used for recording from the depressor nerve 5r1 and the levator nerve 6Br4. All other peripheral nerves were cut close to the ganglion neuropil to block sensory afferents. **(B)** Left: A view from above of the whole-chain preparation during a recording session using seven suction electrodes, and the experimental setup. Right: A ventral view of the whole-chain preparation **(C)** Simultaneous recording of pilocarpine-induced activity of seven motor nerves. The illustration beside each recording trace denotes the identity of the recorded motor nerve: from top to bottom—prothoracic, mesothoracic, and metathoracic ganglion. Red and blue denote depressor and levator MNs, respectively.

Cockroaches were anesthetized with CO_2_ before being fixed to a Sylgard-coated plate ventral side up (Dow Corning 184 Sylgard Silicone Elastomer, Michigan, USA), using minute pins. A ventral longitudinal cut was made, and the entire digestive tract was then gently removed. The head capsule was opened and the desired parts of the central nervous system—isolated thoracic ganglia or a ganglia chain comprising the SEG and the three thoracic ganglia—were dissected out from the cockroach together with their peripheral nerves and main trachea intact, and fixed in a clean Sylgard-coated Petri dish, filled with cockroach saline (58). Levator nerves (6Br4) and depressor nerves (51r) were retained intact while all other peripheral nerves were cut close to their origin (see illustration in Figure 1A). Air was supplied to the ganglia by teasing open the tracheae at the surface of the saline to prevent hypoxia, which is known to be detrimental to thoracic MNs (59). Simultaneous extracellular recordings were conducted using self-fabricated suction electrodes placed on levator and depressor nerves—four for each isolated ganglion or 4–7 electrodes for the whole-chain preparation. The preparations were stimulated by a final concentration of 1^*^10^−5^M pilocarpine (pilocarpine-HCl 99%, Sigma Aldrich, St Louis, MO, USA), freshly prepared in cockroach saline, and bath applied 15 min before recording onset. Motoneuron (MN) activity was acquired using two four-channel differential amplifiers (Model 1700, A-M Systems, USA) and Axon Digidata 1440A digitizer, played in real-time on a PC using Axo-Scope software (Molecular Devices, Sunnyvale, CA, USA). Signals were processed with DataView (W.J. Heitler, University of St. Andrews, Scotland) and MATLAB R2017a (The MathWorks Inc., Massachusetts, USA) with CircStat toolbox (60). For linear statistics and graphs we used Prism 8 (GraphPad Software, San Diego, California USA). Circular graphs were generated using Oriana 4 (Kovach computing services). The preparation and experimental setup are presented in Figures 1A,B.

### Signal Processing and Data Analyses

Ten minute recording bouts were analyzed (see Figure 1C for example of a short recording segment). Threshold spike detection generated event traces of fast and slow depressor MNs, and of levator MNs 5–12 (levator activity mostly comprised MNs 5 and 6). Data were analyzed for the MNs' firing patterns and for the coordination between MNs. Two parameters, *Rhythmicity* and *Burstiness*, describe the dynamic firing pattern of the investigated MNs: *Rhythmicity* is the consistency of the phase relations between time points separated by an interval. Here we calculate the lag coherence between two epochs of the analyzed signal as a measure of rhythmicity, following Fransen et al. (61). In short, the most prominent frequency in the Fourier transformed recording bout was identified, and the original signal was fragmented into adjacent, equal length, non-overlapping epochs of 5 cycles of this frequency (e g., for 0.5 Hz each epoch's length was 10 s). The Fourier coefficient of each epoch was calculated by Fourier transforming the Hanning-tapered signal. Each coefficient is a vector in the complex plain. The vector's angle is the phase relative to the positive horizontal axis and its length is the amplitude. We then calculated for each pair of adjacent epochs the product of $F{\left(x_{n}\right)}_{k}\;F{\left(x_{n+1}\right)}_{k}^{H}$, where F(X) denotes the Fourier transform of the signal X(n). The signals X(n) (for *n* = 1.N) are ordered equal length epochs (5 cycles of the prominent frequency) that were cut from the original 10 min signal. k is the kth Fourier coefficient. H denotes the Hermitian transpose. The results were summed over all epoch pairs (equation 1 numerator). The final sum was then averaged with the number of epochs, to give the consistency of phase relations. Last, the outcome was normalized by the average amplitude in all epochs, to eliminate the dependency on amplitude in favor of the pure measure of rhythmicity, which is valued between 0 (arhythmic) and 1 (perfect rhythmicity), as depicted by equation 1:


$$\begin{array}{l} Rhythmicity\;\left(k\right)=\left|\frac{\sum_{n=1}^{N-1}F{\left(x_{n}\right)}_{k}\;F{\left(x_{n+1}\right)}_{k}^{H}}{\sqrt{\left(\sum_{n=1}^{N-1}|F{\left(x_{n}\right)}_{k}{|}^{2}\;\right)\;\left(\sum_{n=1}^{N-1}|F{\left(x_{n+1}\right)}_{k}{|}^{2}\;\right)}}\right| \end{array}$$

**Figure 2:**
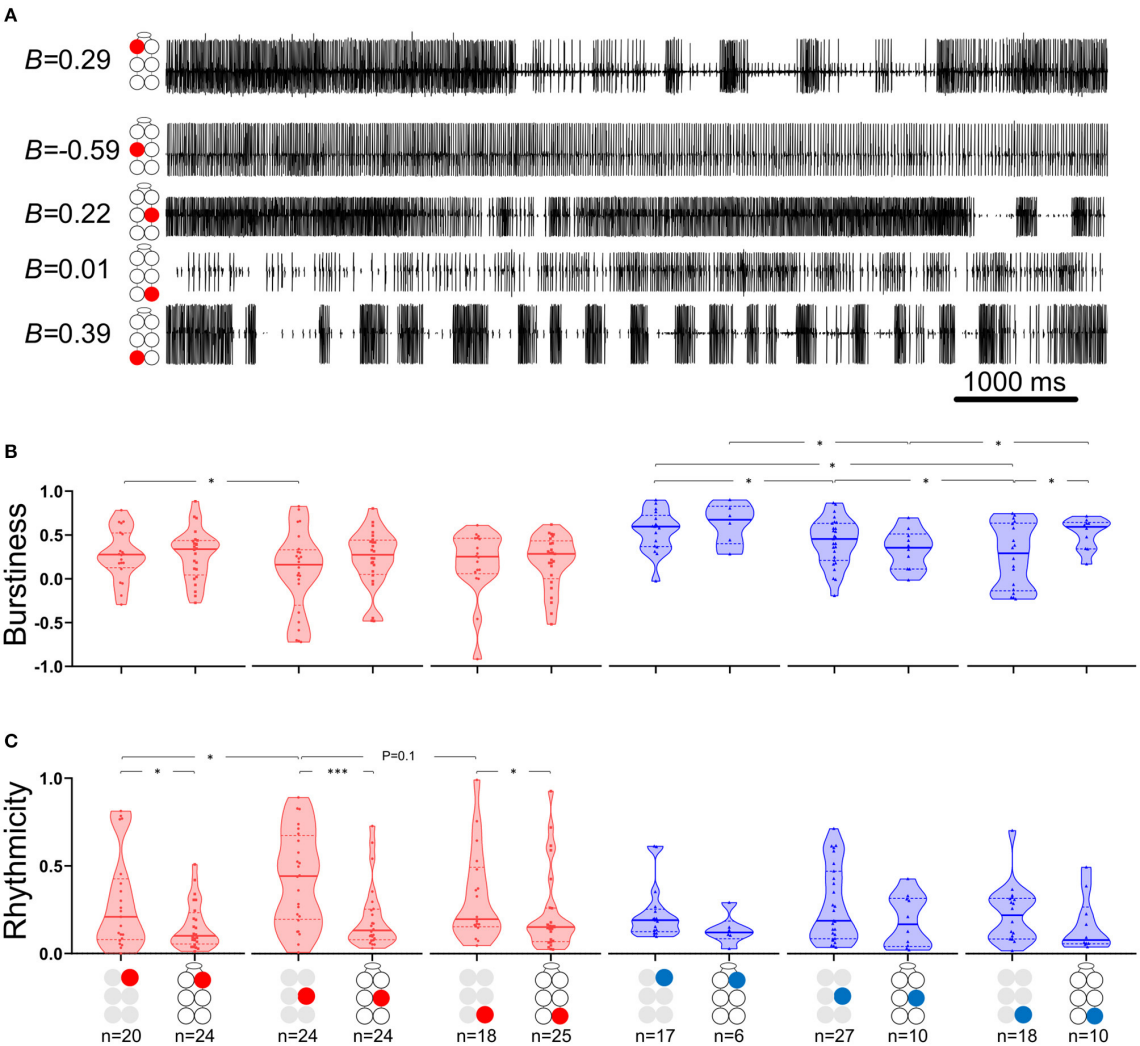
Burstiness and Rhythmicity of MNs in isolated ganglia. Gray and black illustrations represent depressor (red) or levator (blue) MNs from isolated and whole-chain preparations, respectively. Horizontal lines in the violine plots indicate the median (solid) and interquartile range (dash). ^*^,^***^p < 0.05, 0.001. **(A)** A simultaneous recording of five depressor nerves. Burstiness calculated for each trace is presented next to the illustration of the MN identity. Negative and positive burstiness represent tonic and bursting firing, respectively, while zero burstiness represent a poison distribution of interspike intervals. **(B)** Burstiness: Between isolated ganglia: the mesothoracic depressor's burstiness is lower and more variable than that of its prothoracic homolog; burstiness of levator MNs satisfies prothoracic > mesothoracic > metathoracic. The metathoracic levator burstiness is also more variable than that of its homologs. Between connected ganglia: the mesothoracic levator is less bursty than its neighboring levators. Between preparations: Intersegmental connectivity affects the burstiness of only the metathoracic levator, which presents greater and less variable burstiness in the presence of intersegmental coupling. **(C)** Rhythmicity: Between isolated ganglia: The mesothoracic depressor rhythmicity is greater than that of its homologs in neighboring ganglia. Between preparations: All the MNs showed greater rhythmicity in the isolated preparations, although this was significant only for the depressor MNs.

*Burstiness*: bursts are short periods of intense activity followed by periods of inactivity/lesser activity. Burstiness is calculated from the distribution of interspike intervals, and is valued between −1 and 1 (62). B = 1 is a purely bursty signal, B = 0 is neutral (Poisson distribution of interspike intervals), and B = −1 is a completely regular (tonic) signal, as depicted by equation 2:


$$\begin{array}{l} B=\;\left(\sigma_{T}\;-m_{T}\right)\;/\;\left(\sigma_{T}\;+\;m_{T}\right) \end{array}$$

where B = burstiness, σ is the standard deviation of interspike intervals and *m* is the mean interspike interval. Figure 2A presents the burstiness of five MNs in a whole-chain preparation. The coordination between MNs was analyzed by a way of cross-spectrum analysis to assess the *coherence* and *phase-relations* between two event traces (63, 64). Event traces were first bandpass filtered for 0.05–10 Hz, to exclude most of the non-bursting activity. This bandpass is 20-fold wider than usually seen and analyzed in similar *in vitro* insect preparations. This relatively fast activity could be due to greater excitability of the cockroach motor centers, which also manifests in the 10–50-fold lower concentration of pilocarpine needed to induce long-lasting rhythmic activity in the cockroach preparation in comparison to locust (51, 65), stick insect (42), and moth (66). Additional parameters comprised the *Coherence* and *Phase-relations* of two signals. *Coherence* is defined by the IEEE Standard Dictionary (67) as “the correlation between electromagnetic fields at points which are separated in space or in time, or both.” It is the measure of the causal relationship between two signals in the presence of other signals and will always satisfy 0 ≤ Coherence ≤ 1. Coherence is used to measure mono-synaptic iso-frequency (i.e., “direct”) coupling between elements in a network (68); and is used here to assess the association between activity recorded from two MNs within an isolated ganglion, but not the whole-chain preparation. Confidence intervals of coherence were calculated following Rosenberg et al. (69). Here, the coherence is normalized to the highest value we calculated from our analyzed data. *Phase-relations* (phase) measures the relative timing of activity in one MN with respect to the activity of another MN. Here, phases were further processed for analysis only if their corresponding coherence was statistically significant (i.e., significant phases). The significant phases were averaged to give a single value of phase for each pair of MNs in each experiment. The products of different experiments were grouped to enable comparisons between different pairs of MNs. Hereafter phase refers only to significant phase. Two additional related parameters were calculated. The first, *Coupling strength* (CS), was calculated in order to also account for the variability of phase. CS is calculated by multiplying the length of vector of the phase by the mean coherence. Unlike the phase-independent coherence, CS also considers the phase-lock to produce a measurement of functional coupling. This distinction is important, since pairs of network units can present high or low coherence, regardless of the consistency of their phase. The second parameter is the *Synchronization index* (SI), which is a combined measure of the mean and variability of the phases. The linear SI (as opposed to the circular phase) represents the type of coordination (in-phase or antiphase) that a pair of MNs demonstrates, and the phase-lock. In brief, SI is the product of projecting the mean phase vector onto the 0–180 axis. The calculation is based on Knebel et al. [(51) and references within] but differs in that SI was calculated separately here for each experiment, to enable statistics and comparisons. The use of the linear SI instead of the circular phase also enabled the use of linear statistics instead of the relatively limited circular statistics. SI is defined between 1 (perfect in-phase) to −1 (perfect antiphase) with ±5% confidence intervals of ±0.081 (see Supplementary Figure 1 for more details). All data are presented as Mean ± Confidence-Intervals (CI) unless noted otherwise. Detailed data tables are presented in the Supplementary Materials.

### Terminology and Abbreviations

In order to correctly identify the MN pairs referred to here, each MN is coded as followed: side of the body (right/left, R/L)-thoracic segment (1,2,3 for pro-, meso-, and meta-thorax, respectively)-function (levator/depressor, Lev/Dep). For example: R2Dep-L2Lev represents the pair comprising the right mesothoracic depressor and left mesothoracic levator (see illustration in Figure 1A). In addition, pairs comprising two MNs performing the same function are referred to as “homogenous.” Moreover, we use the terms “in-phase” and “antiphase,” which correspond to phase relations of 0 and 180°, to describe a range of phase relations according to their proximity to the ideal values noted above: in-phase between 270 and 90° and antiphase between 90 and 270°.

## Results

Before the application of pilocarpine, we observed either no activity or a motor output characterized by low burstiness, which usually did not persist for more than a few minutes before the preparation became quiescent. The following results are all from pilocarpine-stimulated preparations (see reference to this point in the Discussion).

### Isolated Ganglia Preparations

Each thoracic ganglion controls a pair of contralateral legs. The pairs differ in their size, shape, and function. These differences suggest that the underlying neural control also differ. To investigate this, we characterized the burstiness and rhythmicity of the motor output recorded from homolog depressor and levator MNs in the three thoracic ganglia. In addition, we performed a comparative analysis of the temporal relations between motoneuron activity within the isolated ganglia: frequency, coherence, phase, the type of coordination (in-phase or antiphase), and the coupling strength. We further tested for frequency-dependent differences in the calculated parameters.

#### Levators Burstiness, but Not Rhythmicity, Varies Between Ganglia. Depressors Present the Opposite

The data presented in Figure 2 and in Supplementary Tables 1, 2 describe the burstiness (Figure 2B) and rhythmicity (Figure 2C) of homolog MNs in the isolated pro-, meso-, and meta-thoracic ganglion. Burstiness of the R2Dep was lower than that of R1Dep (Welch's *t*-test, *p* < 0.05), and more variable than in both R1Dep and R3Dep (Brown-Forsythe, *p* < 0.05). Levator burstiness satisfied prothoracic > mesothoracic > metathoracic (Brown-Forsythe Anova, *p* < 0.05). Surprisingly, burstiness was not correlated with rhythmicity (Spearman's or Pearson's correlation, *p* > 0.05). R2Dep showed greater rhythmicity than both R1Dep and, although not statistically significant, R3Dep (Mann-Whitney, *p* < 0.05 and *p* = 0.1, respectively). Levator MNs rhythmicity was similar in all the ganglia (*p* > 0.2).

#### Temporal Relations Between Motoneurons in the Isolated Ganglia

Although similar studies of other insect *in-vitro* preparations have shown findings that were obtained from low-frequency motor activity of up to 0.5 Hz (42, 51, 65, 66), our cockroach *in vitro* preparation showed burst frequencies as high as 10 Hz (although 9–10 Hz activity was scarce and mostly uncoordinated). Therefore, we first analyzed a wide range of frequencies and then, following our findings, we limited the range of frequencies for further investigation.

##### Coherence Is Frequency-Dependent Only in the Contiguous Pairs

**Figure 3:**
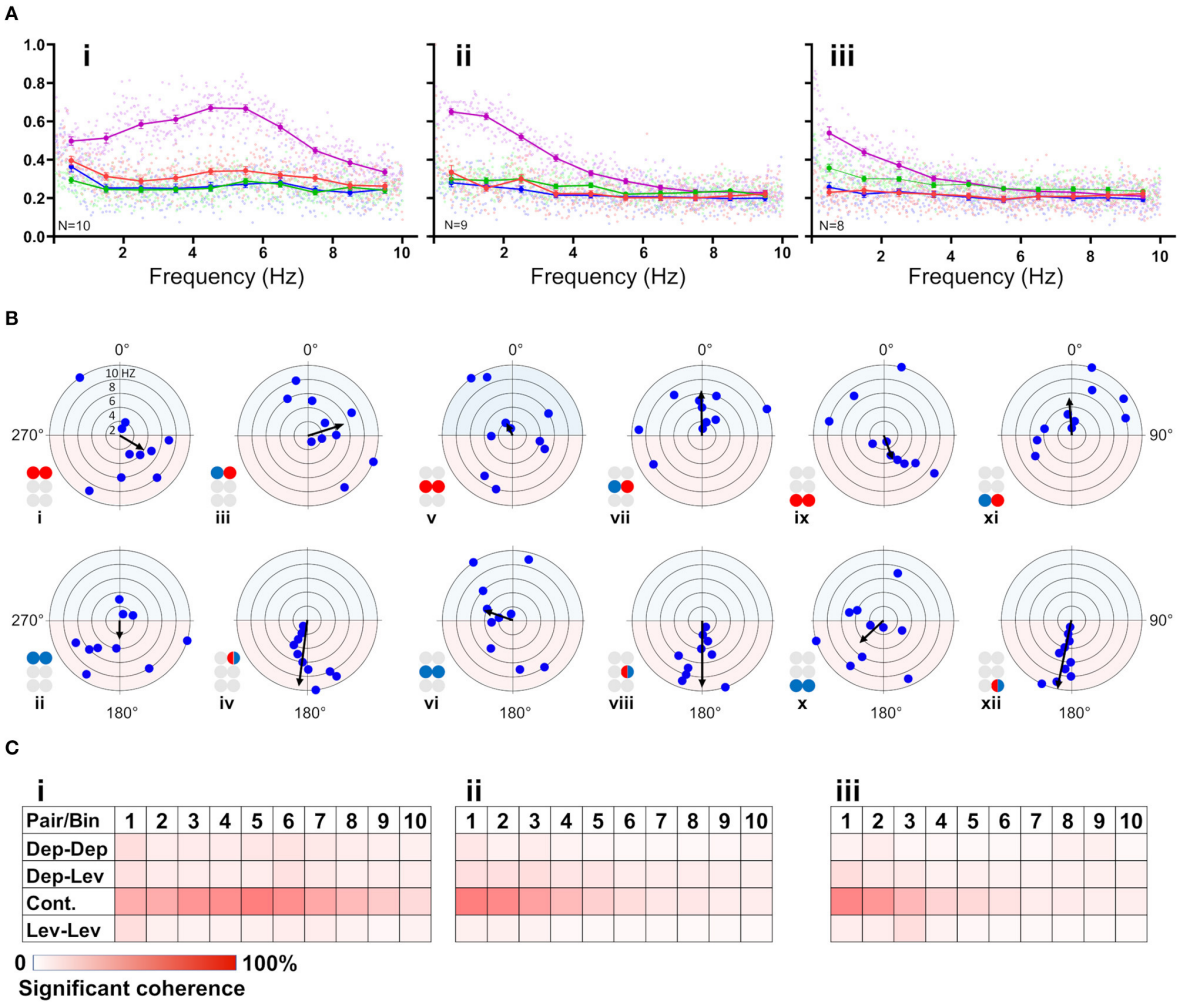
**(A)** Frequency-dependent coherence in the isolated ganglion preparations. Scatter plots and overlying lines are colored by the type of connection: pink for contiguous, red for dep-dep, green for dep-lev, and blue for lev-lev. Overlying lines present the mean ± CI of normalized coherence, at the middle of each bin (0.5, 1.5 Hz…9.5 Hz), for 10 bins of frequencies (bin size = 1 Hz, *n* = 60 samples). Data are normalized with the greatest value of coherence measured in this investigation. The contiguous pairs differ between the ganglia in how their coherence changes with frequency. While the prothoracic contiguous pair maintains greater coherence than the prothoracic contralateral pairs, those in the mesothoracic and metathoracic ganglia present a trend of decoupling toward the coherence of the contralateral pairs. **(B)** Frequency-dependent phases in the isolated ganglia preparations. The circles illustrated beside each histogram are colored according to the motor nerves function—red and blue for depressor and levator, respectively. Mixed colors denote a pair of levator and depressor within a hemiganglion. The circular-linear plots are pale blue (270° 90°) and red (90° 270°) to represent in-phase and antiphase coordination, respectively. Each point in the plots represents the mean phase of a 1 Hz bin. Grid lines = 2 Hz (detailed at i). Frequency increases with the distance of the point from the center of the plot. The black arrow is the vector of phase, calculated for the entire 10 Hz range of frequencies. The prothoracic homogenous pairs present a bi-phasic frequency-dependent coordination: in-phase at low frequency (<2 Hz and <3 Hz for Dep-Dep and Lev-Lev) and antiphase at greater frequencies. Mesothoracic Dep-Dep and Lev-Lev present in-phase coordination at frequencies <2 Hz like their prothoracic homologs, and an inconsistent, highly variable coordination in greater frequencies. The metathoracic ganglion presents a relatively consistent antiphase coordination in the homogenous pairs. The metathoracic ganglion is unique in that it presents a tripod gait coordination in all pairs throughout all the bins. **(C)** Color-tables of significant coherence. Darker red indicates a greater fraction of significant values of coherence out of the total values calculated in each bin (60 per bin).

First, the coherence between paired MNs was calculated and filtered to include frequencies between 0.05:10 Hz and exclude non-bursting activity (ste*p* = 0.0167 Hz; coherence is presented in Supplementary Table 3). The coherence was then binned in 10 frequency groups: 0.05–1, 1–2 Hz,…9–10 Hz. The first bin comprised two values less than the other bins (values lower than 0.05 Hz). Next, the relations between coherence and frequency were characterized for all possible pairs of MNs in each isolated thoracic ganglion: the contralateral pair of depressors, the contralateral pair of levators, a pair of contralateral depressor and levator, and the contiguous pair (within a hemiganglion) of depressor and levator (hereafter, Dep-Dep, Lev-Lev, Dep-Lev, and contiguous, respectively). The findings are illustrated in Figure 3A. For an in-dept analysis we used two-way ANOVA with repeated measures of the row factor (i.e., frequency) and a Tukey test for *post-hoc*. Our analysis revealed that the contiguous pair had greater mean coherence than that of the corresponding contralateral pairs at frequencies lower than 5 Hz in the metathorax (*p* < 0.01, Figure 3Aiii), and lower than or equal to 7 Hz in the mesothorax (*p* < 0.01, Figure 3Aii); and this was also the case for the prothoracic pair throughout the entire range of frequencies tested (*p* < 0.001, Figure 3Ai). In addition, a Friedman's test calculated based on the mean coherence of each bin, and followed by Dunn's post hoc test, revealed that R3Dep-L3Lev had a greater mean coherence than R3Dep-L3Dep and R3Lev-L3Lev; while in the mesothoracic ganglion the coherence was similar for all three contralateral pairs; and in the prothoracic ganglion R1Dep-L1Dep had greater mean coherence than R1Dep-L1Lev (Dunn's, *p* < 0.05). A second two-way ANOVA was calculated in order to examine the differences between pairs and between ganglia. R1Dep-R1Lev and R1Dep-L1Dep mean coherence was found to be greater than their homologs in the other ganglia (Tukey, *p* < 0.01). In addition, R3Dep-L3Lev was found to be greater than R2Dep-L2Lev. Moreover, the R1Dep-R1Lev and R1Lev-L1Lev showed greater mean coherence than their homologs in the other ganglia (Tukey, *p* < 0.05; data are presented in Supplementary Figure 2 and Supplementary Table 3). Another difference between the ganglia is seen in the way the coherence of the contiguous pairs underwent change with frequency. R1Dep-R1Lev showed relatively high coherence throughout most of the investigated frequency band, with a wide parabolic distribution that peaks at about 5 Hz, while R2Dep-R2Lev peaks at about 0.5 Hz and sharply decreases above 2 Hz, and R3Dep-R3Lev decreases from the first indexed frequency (0.05 Hz) and onward. These findings may indicate that a strong intra-hemiganglion coherence is especially important for the appropriate function of the prothoracic control network, at all frequencies.

##### Phase-Relations Are Frequency-Dependent for Homogenous Prothoracic and Mesothoracic Pairs, and Frequency-Independent for Heterogenous and All Metathoracic Pairs

Frequency-dependent coherence suggested that phase might also vary with frequency. This had implications for our choice of the range of frequencies to be analyzed here, as well as for the possible interpretations of (partial findings from) previous studies. To investigate this, phase was calculated and binned in ten frequency groups: 0.05–1, 1–2 Hz,…9–10 Hz. Mean phase was calculated for each preparation separately, for each of the 10 group of frequencies. Figure 3B and Supplementary Table 4 present the mean calculated for all 20 preparations (*N* = 20). In general, the contiguous pairs displayed a consistent and frequency-independent antiphase coordination throughout all the bins, in all ganglia (Figure 3Biv,viii,xii). Moreover, the Dep-Lev pairs displayed a frequency-independent phase with lower variability than their corresponding Dep-Dep and Lev-Lev pairs (Figure 3Biii,vii,xi). R1Dep-L1Dep and R1Lev-L1Lev presented bi-phasic coordination: in-phase up to 2 and 3 Hz (respectively), and antiphase above it (Figure 3Bi,ii; Watson-Williams, *p* < 0.05). Likewise, R2Dep-L2Dep and R2Lev-L2Lev showed in-phase coordination up to 2 Hz, although weaker for R2Lev-L2Lev (Figure 3Bv,vi). The coordination of both pairs became inconsistent at higher frequencies. In contrast, R3Dep-L3Dep and R3Lev-L3Lev presented an overall frequency-independent antiphase coordination (Figure 3Bix,x). In addition, we found a frequency-dependent variability in the number of significant phases that were calculated for each of the frequency bins, mostly in favor of the lower frequencies (Figure 3C). This phenomenon is predominantly linear in the meso- and meta-thoracic ganglia (Figure 3Cii,iii), and parabolic in the prothoracic ganglia (Figure 3Ci), a pattern that corresponds to the frequency-dependent variability of coherence of the different contiguous pairs. The frequency-dependent decrease in coherence manifests as fewer bursts and more transient spikes in the medium-to-high range frequencies. At the highest investigated frequencies 9 and 10 Hz, the simultaneous bursting of different MNs was scarce, mostly with below-threshold coherence, and with an inconsistent phase. Since the major share of significant phases (i.e., eligible for analysis) was sampled between 0.05 and 3 Hz, and includes the changes we observed at 2 Hz in some of the pairs, and also to enable a better comparison with studies of other *in-vitro* insect models, as noted above, we chose to focus on the frequency band 0.05–3 Hz for the further analyses of the isolated ganglion preparations.

##### Contralateral Coordination Differs Between the Isolated Ganglia, and Is Functional Only in the Metathoracic Ganglion

**Figure 4:**
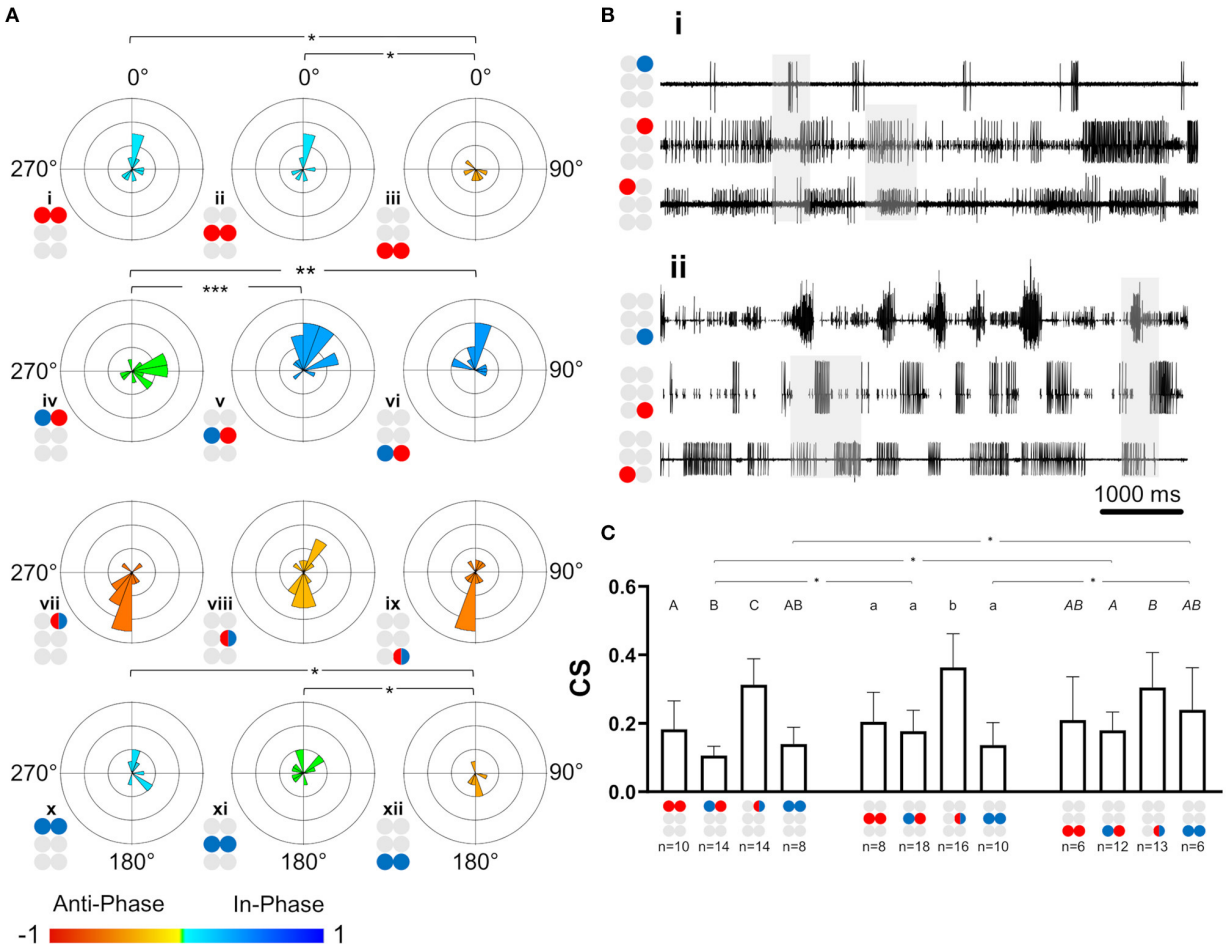
Illustrations of circles are colored according to the motor nerves function—red and blue for depressor and levator, respectively. Mixed colors denote a pair of levator and depressor within a hemiganglion. **(A)** Synchronization in the isolated ganglia: Phase histogram color represents the synchronization index, which is defined between 1 (perfect in-phase, blue), 0 ± 0.08 (neutral, green) and −1 (perfect antiphase, red), as seen in the color bar. Grid lines = 2. ^*^,^**^,^***^p < 0.05, 0.01, 0.001. In all ganglia the coordination between the coxa-trochanter joint antagonistic MNs is antiphase. In contrast, the coordination between contralateral MNs differs between the ganglia, and shows tripod-like phases only in the metathoracic ganglion. **(B)** Rhythmic activity in the isolated prothoracic (i) and metathoracic (ii) ganglia. Gray shade is used for emphasizing the following findings. The contralateral depressors (2nd and 3rd traces in each panel) present in-phase coordination in the prothoracic ganglion, and antiphase coordination in the metathoracic ganglion. The contralateral Dep-Lev (1st and 3rd traces in each panel) present a mix, variable, coordination in the prothoracic ganglion, and a tripod-appropriate in-phase coordination in the metathoracic ganglion. In contrast to these pairs, the contiguous pairs (1st and 2nd traces in each panel) present antiphase coordination that corresponds to fictive stepping in both ganglia. **(C)** Coupling strength in the isolated ganglia preparations. Data are presented as mean + CI. Sample sizes are the same for the corresponding histograms. Letters above the bars indicate for the significance of the difference between the bars (majuscule, minuscule, and *italic* for pro-, meso- and meta-thoracic ganglia). Bars that share a letter are not significantly different (*p* > 0.05). The metathoracic contralateral levators are coupled stronger than their prothoracic and mesothoracic homologs, while the contralateral Dep-Lev pair in the prothorax is weakly coupled in comparison to the mesothoracic and metathoracic homologs.

After the data had been filtered for the appropriate frequency band (0.05–3 Hz), the intra-ganglionic coordination was characterized. A synchronization index (SI) was calculated to give a combined, linear, and comparable measure of coordination and its strength, for the frequency range comprising most of our data. Data are illustrated in Figure 4A and detailed in Supplementary Table 5. Significance of differences was calculated using a Mann-Whitney test with a Bonferroni correction for two comparisons. Differences in SI were found between contralateral pairs. For Dep-Dep pairs, R3Dep-L3Dep antiphase coordination (Figure 4Aiii) significantly differed from the in-phase coordination found in R1Dep-L1Dep, R2Dep-L2Dep (Figure 4Ai,ii,iii. SI = −0.483 ± 0.34, 0.125 ± 0.36, and 0.097 ± 0.49, respectively; *p* < 0.025). For Dep-Lev pairs, R1Dep-L1Lev showed neutral synchronization (i.e., between in-phase and antiphase: mean ± CI = 0 ± 0.08), in contrast to in-phase coordination in R2Dep-L2Lev and R3Dep-L3Lev (SI = −0.062 ± 0.1, 0.355 ± 0.16 and 0.396 ± 0.21, respectively; *p* < 0.01, Figure 4A). These differences are demonstrated in the recordings presented in Figure 4B. R1Dep and L1Dep were in-phase coordinated, and R1Dep and L1Lev coordination was inconsistent (Figure 4Bii), in contrast to the antiphase coordination of R3Dep and L3Dep, and the consistent in-phase coordination of R3Dep and L3Lev (Figure 4Bii). Last, the prothoracic and mesothoracic Lev-Lev synchronization was found to be in-phase and neutral, respectively, unlike the significant difference in the antiphase synchronization found in the metathoracic ganglion (SI = 0.122 ± 0.3, 0.046 ± 0.25 and −0.418 ± 0.38, respectively; *p* < 0.025, Figure 4Ax,xi,xii). Overall, only the isolated metathoracic ganglion showed an intra-ganglion coordination that corresponded to that expected for the tripod gait (Figure 4Aiii,vi,xii).

##### Coupling of Dep-Lev and Lev-Lev Is Ganglion-Specific

Following establishment of the type of coordination, the strength of the central coupling (CS) was examined (data are detailed in Supplementary Table 6). As presented in Figure 4C, Dep-Dep pairs were similarly coupled in all ganglia. This was also the case for the homolog contiguous pairs, which also had greater CS in comparison to the other pairs of MNs in the prothoracic and mesothoracic ganglia (*t*-test or Mann-Whitney with Bonferroni correction, *p* < 0.017). In contrast, the comparison between ganglia revealed two significant differences: Dep-Lev coupling was weaker in the prothoracic ganglion and Lev-Lev coupling was stronger in the metathoracic ganglion, in comparison to their homologs in the other ganglia (Mann-Whitney with Bonferroni correction *p* < 0.025). These findings provide further evidence that the intrinsic local networks themselves are not identical and that their endogenous connectivity and/or the synaptic strength of the connections, are designed to enable different functionality.

### The Whole-Chain Preparation

**Figure 5:**
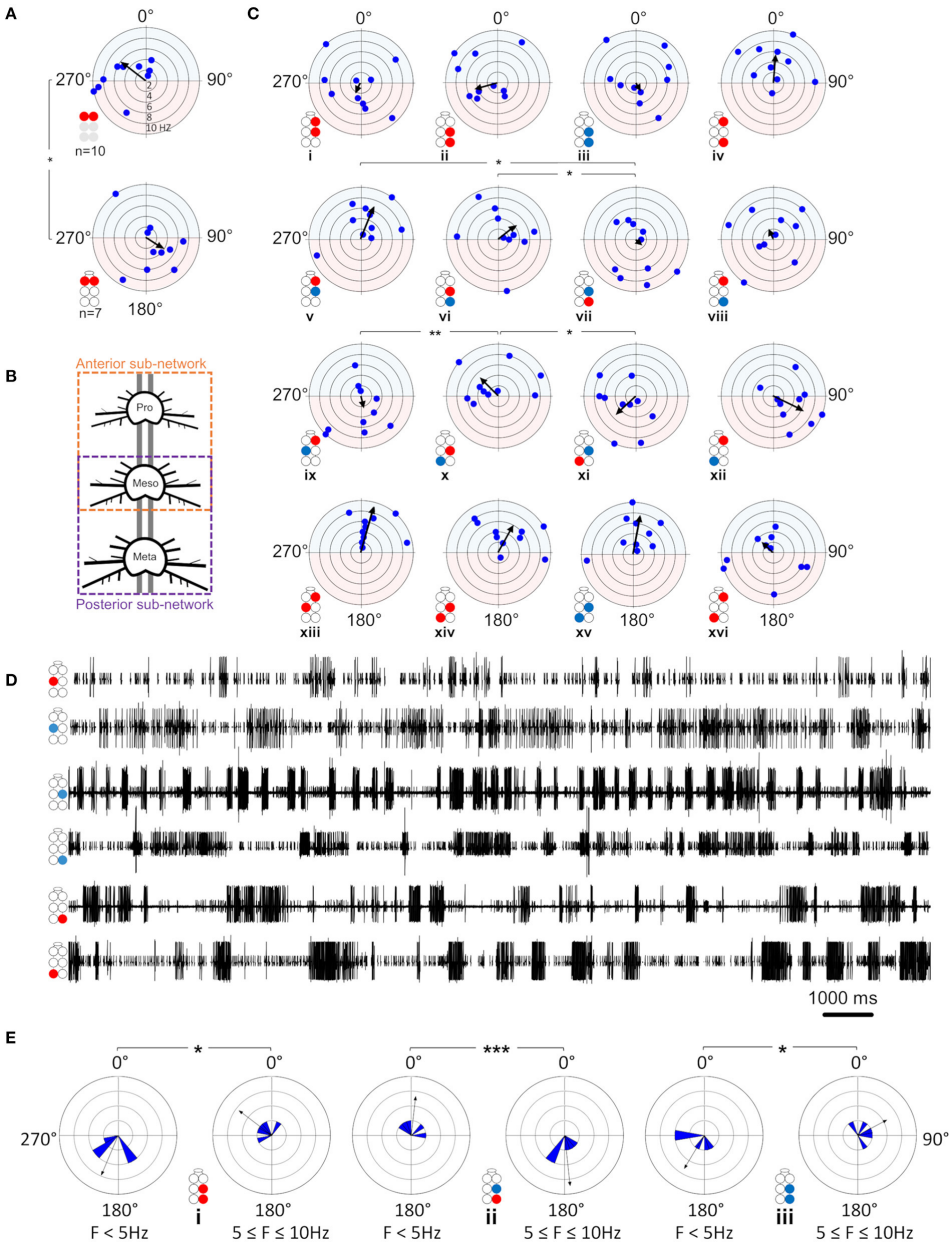
Illustrations of circles are colored according to the motor nerves function—red and blue for depressor and levator, respectively. Illustrations with gray circles represent the isolated preparation and illustrations with black empty circles represent the whole-chain preparation. The circular-linear plots are pale blue (270° 90°) and red (90° 270°) to represent in-phase and antiphase coordination, respectively. Grid lines = 2 Hz (detailed at **A**, top plot). Arrow-vector of phases. ^*^,^**^p < 0.05, 0.01 **(A)** Interganglion connectivity affects the coordination of the prothoracic depressors. The prothoracic pair of depressors presents a similar in-phase coordination at frequency <2 Hz in both isolated (top) and whole-chain (bottom) preparations, and different types of coordination at greater frequencies. The other six intraganglion pairs of MNs that were compared between the preparations were found to present non-significant differences in phases at the different frequencies. **(B)** Illustration of the division of the whole-chain preparation into two sub-networks- anterior and posterior. **(C)** Frequency dependent interganglia phases. Left column- anterior sub-network, middle columns- posterior sub-network, right column- between non-neighboring ganglia. Rows from top to bottom: ipsilateral homogenous, ipsilateral mixed, diagonal mixed, and diagonal homogenous. Significant differences between the sub-networks were found only in the mixed Dep-Lev ipsilateral and diagonal pairs, and not in the homogenous pairs. In the anterior sub-network, R1Dep-R2Dep (i) and R1Dep-L2Lev (ix) which alternate during tripod locomotion changed from in-phase to antiphase coordination at frequencies > 2 Hz, while R1Dep-L2Dep (xiii) and R1Dep-R2Lev (v), which fire in-phase during tripod locomotion, demonstrated a frequency-independent coordination. In the posterior sub-network, asymmetries were found between reciprocal mixed pairs in the ipsilateral [R2Dep-R3Lev (vi) and R2Lev-R3Dep (vii)], and the diagonal [R2Dep-L3Lev (x) and R2Lev-L3Dep (xi)] pathways. **(D)** Rhythmic activity in the posterior sub-network. Both contiguous pairs show antiphase coordination (1st and 2nd traces, and 4th and 5th traces). The ipsilateral pairs R2Lev-R3Lev (3rd and 4th traces), and L2Dep-L3Dep (1st and 6th traces) show weaker coupling strength and less rigid phase relations than the intraganglion pairs. **(E)** Frequency-dependent coordination inversion of ipsilateral pairs in the posterior sub-network. Grid line = 1. The phase histograms illustrate three pairs switching from tripod-like coordination at low frequencies into a faster gait at higher frequencies in which two ipsilateral legs can simultaneously perform the aerial phase.

With the exception of coherence, the same set of parameters and analyses used for the investigation of the single isolated ganglion preparations was also used for characterization of the activity and coordination of the depressor-levator network in an intact chain of the thoracic and subesophageal ganglia. The overall connectivity network of this preparation potentially comprises 36 different pairs of MNs, for which recording and analyzing a reliable sample is an overwhelming task. A total of seven intra-ganglion connections were investigated in the whole-chain preparation: R1Dep-L1Dep, R2Dep-L2Dep, R3Dep-L3Dep, R2Dep-L2Lev, R3Dep-L3Lev, R2Dep-R2Lev, and R3Dep-R3Lev. We then focused on 16 interganglia connections: six Dep-Dep, eight Dep-Lev, and two Lev-Lev, detailed in Figure 5C. The network was divided into two sub-networks: anterior for the prothoracic-mesothoracic connections and posterior for the mesothoracic-metathoracic connections (see Figure 5B for illustration). This was done in order to examine the differences and similarities between homolog connections in the two sub-networks. The calculated phases between MNs are referred to as tripod-gait-appropriate if they corresponded to those recorded (or could be recorded) in the intact walking insect. Here we focused mostly on mesothoracic-metathoracic pairs of MNs (8 pairs), rather than on prothoracic-mesothoracic or prothoracic-metathoracic ones (4 pairs each), because most of the previously published relevant research refers to mesothoracic and metathoracic MNs (38, 48, 70–72). Moreover, we chose to focus our investigation on connections between the depressor MNs (all 9 pairs), which again enabled comparison to the ample related previous research (46, 51, 54, 55, 73).

#### Levators Burstiness, but Not Rhythmicity, Differ Between Preparations and Between Ganglia, While Depressors Present the Opposite

The effects of intersegmental connectivity on MNs burstiness and rhythmicity were studied by comparing the isolated with the whole-chain preparations. As can be seen in Figure 2B and Supplementary Table 1, the intersegmental connectivity had a statistically significant effect on the burstiness of only one motor nerve—the metathoracic levator, which had twice the mean burstiness, and half the variability, in the presence of intersegmental connectivity (mean ± SD for isolated and whole-chain preparations: 0.51 ± 0.18 > 0.25 ± 0.36, *n* = 18 and 10 accordingly; Welch's *t*-test, *p* < 0.01). This was followed by a comparison between homolog MNs in the whole-chain preparation, which revealed that the mesothoracic levator was less bursty than the other levators (Welch's *t*-test, *p* < 0.05). Figure 2C and Supplementary Table 2 present a similar analysis of rhythmicity, demonstrating that it was consistently greater in the isolated ganglion, although the differences were statistically significant only for the depressor MNs (Mann-Whitney, *p* < 0.05). Moreover, the variability in rhythmicity of the prothoracic depressor MN was found to be lower than that of its meso- and meta-thoracic homologs; while for the levator the difference was significant only in comparison to its mesothoracic homolog (Brown-Forsythe, *p* < 0.05).

#### Temporal Relations Between MNs in the Whole-Chain Preparation

The relations between phase and frequency were studied in the whole-chain preparation for a frequency band of 0.05–10 Hz (data are given in Supplementary Table 7). First, the seven intra-ganglion connections were studied and compared between the isolated and whole-chain preparations. Overall, 6 out of 7 intra-ganglion connections showed similar phase relations in both preparation types throughout the entire frequency range (Supplementary Figure 3), suggesting that their coordination is not significantly influenced by intersegmental or SEG inputs. In contrast to the other pairs, R1Dep-L1Dep fired in-phase at low frequencies and in antiphase at frequencies > 2 Hz in the isolated preparation, as opposed to the consistent in-phase coordination it showed in the whole-chain preparation (Φ_isolated_ = 121.2 ± 77.8, Φ_whole−chain_ = 306.7 ± 58.2, *p* < 0.001, Figure 5A). In addition, although R2Dep-L2Dep had an overall similar mean phase in both preparations, the vector length of the mean phases was 3-fold greater in the whole-chain preparation (R = 0.185 and 0.563 for isolated and whole-chain preparations, respectively), suggesting a stabilizing input to the mesothoracic ganglion.

##### Frequency-Dependent Phase-Relations Differ Substantially Between the Anterior and Posterior Sub-networks

As noted above, the network was divided into two sub-networks: anterior and posterior (Figure 5B). Frequency-dependent phase data for four pairs recorded from the anterior sub-network are presented in Figure 5C. R1Dep-R2Lev and R1Dep-L2Dep maintained a tripod-gait-appropriate in-phase coordination throughout the examined frequency range (Figure 5Cv,xiii, respectively). The latter is in accordance with a finding from locusts that a front leg and its diagonal middle leg are always strictly coordinated in phase (74). In contrast, R1Dep-R2Dep and R1Dep-L2Lev, which showed an antiphase coordination during tripod locomotion, had in-phase coordination below 2 Hz, and a robust antiphase coordination only at greater frequencies (Figure 5Ci,ix, respectively). This pattern corresponds to that seen in the prothoracic and mesothoracic Dep-Dep and Lev-Lev pairs in the isolated ganglion preparations (Figure 3Bi,ii,v,vi). In addition, eight pairs were recorded from the posterior sub-network. In contrast to their anterior sub-network homologs, R3Dep-L3Dep and R3Lev-L3Lev showed a tripod-appropriate antiphase coordination from the lowest end of the frequency band, and roughly to its middle (5 and 4 Hz for R2Dep-R3Dep and R2Lev-R3Lev, Figure 5Cii,iii, accordingly). Similarly, the ipsilateral mixed pair R2Lev-R3Dep had in-phase coordination only at frequencies <5 Hz (Figure 5Cvii). These findings are also demonstrated in the recording sample of mostly low-frequency activity (<5 Hz) in the posterior sub-network which is presented in Figure 5D. A comparison between calculated phases from the two halves of the frequency range (lower and higher, all phases are given in Supplementary Figure 4) further support these findings. R2Dep-R3Dep and R3Lev-L3Lev practically inverted from predominantly antiphase to in-phase coordination with the increase in frequency, while in R2Lev-R3Dep the change was the opposite (R2Dep-R3Dep: Φ_<5Hz_ = 202.8° ± 45.4, Φ_5−10*Hz*_ = 307.1° ± 48.9, *p* < 0.05, Figure 5Ei; R2Lev-R3Lev: Φ_<5Hz_ =211° ± 54.2, Φ_5−10*Hz*_ 62.1° ± 62.7, *p* = 0.01, Figure 5Eii; R2Lev-R3Dep: Φ_<5Hz_ = 6.5° ± 51.7, Φ_5−10*Hz*_ = 173.9° ± 31.8, *p* < 0.001, Figure 5Eiii). This finding may suggest that the posterior sub-network is wired to generate fast locomotion (faster than that using the tripod gait) that shows at least a partial overlap in the swing phases of the ipsilateral neighboring legs. Oddly, unlike R2Lev-R3Dep, the reciprocal pair R2Dep-R3Lev showed in-phase coordination throughout the entire frequency band (Φ = 52° ± 70.4, Figure 5Cvi). This asymmetry, along with others, is addressed in the Discussion. Next, we studied the diagonal pairs and found that the homogenous pairs R2Dep-L3Dep and R2Lev-L3Lev were in-phase coordinated regardless of frequency (Figure 5Cxiv,xv, respectively). However, as in the ipsilateral pairs, the mixed diagonal pairs were asymmetrical: while R2Dep-L3Lev had an overall in-phase coordination throughout the frequency band (Φ = 315.2° ± 67, Figure 5Cx), R2Lev-L3Dep had antiphase coordination (Φ = 227.3° ± 65.2, *p* < 0.001, Figure 5Cxi). Finally, we examined the coordination between the prothoracic and metathoracic ganglia. Only R1Dep-L3Dep showed frequency-dependent phases (Φ_<5Hz_ = 334.3° ± 21.3, Φ_5−10*Hz*_ = 182.8° ± 71.2, *p* < 0.01, Figure 5Cxvi). R1Dep-R3Dep had a consistent in-phase coordination (Figure 5Civ), while the two heterogeneous pairs had dysfunctional (i.e., not corresponding to known insect gait) phases (Figure 5Cviii,xii).

##### Tripod-Appropriate Coordination Is Found Only in the Posterior Sub-network

**Figure 6:**
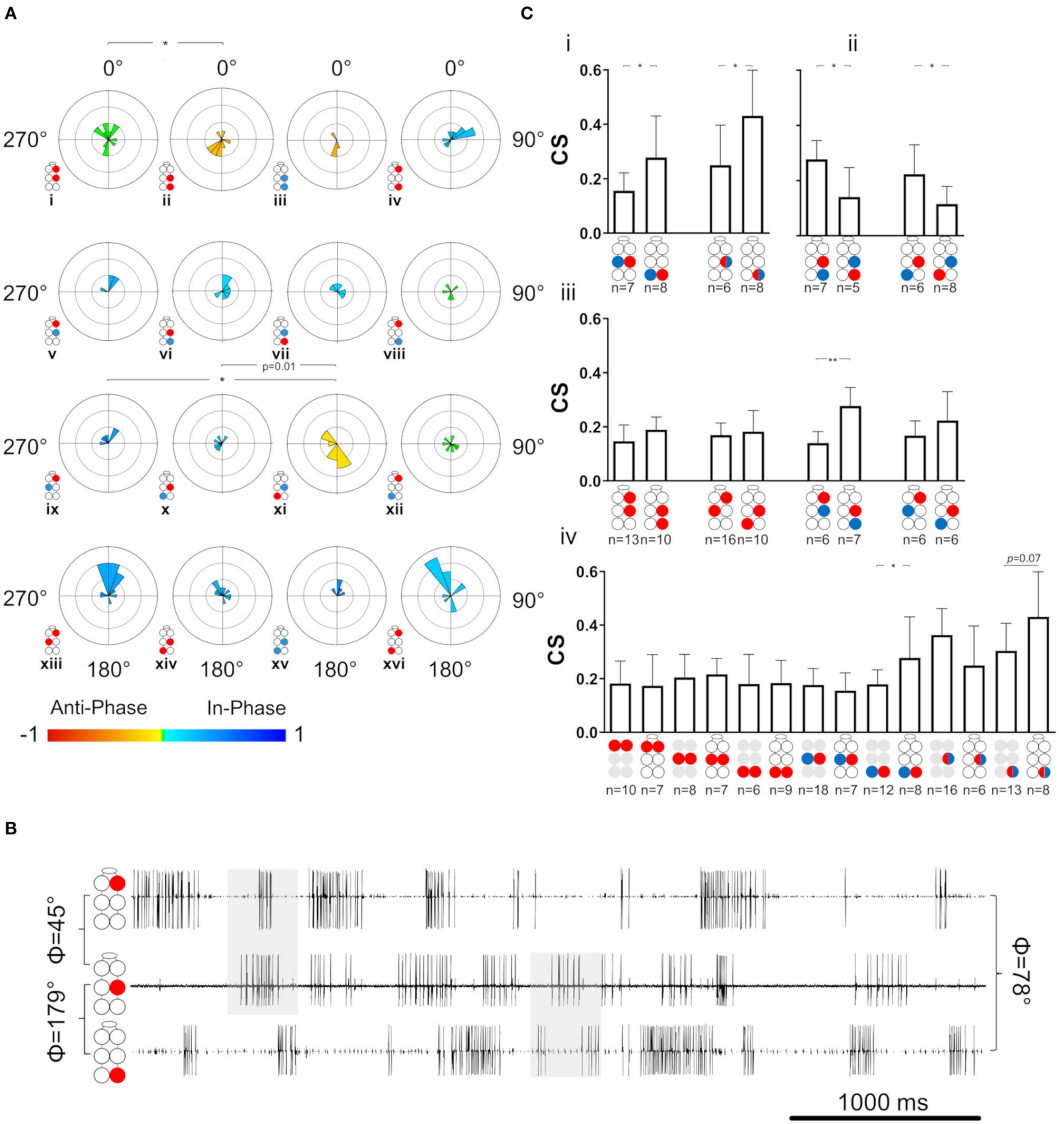
Coordination and coupling in the whole-chain preparation. Illustrations of circles are colored according to the motor nerves function—red and blue for depressor and levator, respectively. Mixed colors denote a pair of levator and depressor within a hemiganglion. Illustrations with gray circles represent the isolated preparation and illustrations with black empty circles represent the whole-chain preparation. **(A)** Synchronization of interganglia connections. Grid lines = 2. ^*^,^**^= *p* < 0.05. 0.01, accordingly. Circular histograms are ordered like the circular plots in Figure 5. The anterior sub-network is tuned to in-phase coordination, while the posterior sub-network presents antiphase coordination in pairs that alternate during tripod locomotion, indicating the dominancy of the metathoracic ganglion in dictating phases for the entire network during fast locomotion. **(B)** Simultaneous activity of the ipsilateral depressor MNs. Top to bottom: right pro-, meso-, and meta-thoracic depressor. Mean phase is presented for the three pairs of MNs. As seen in the examples shaded in gray, R1Dep-R2Dep presents the in-phase coordination that characterize the anterior sub-network, while R2Dep-R3Dep presents the posterior sub-network characteristic antiphase coordination. Although R1Dep-R3Dep presents in-phase coordination, it is less distinct than that of R1Dep-R2Dep. **(C)** Coupling in the whole-chain preparation. Data are presented as mean ± CI. (i) Homolog intraganglion mixed pairs are coupled more strongly in the metathoracic ganglion. (ii) Reciprocal interganglia Dep-Lev pairs are coupled more strongly if their levator MN is metathoracic. (iii) Stronger posterior sub-network coupling is consistent but significant only for the ipsilateral Dep-Lev pair (iv) A comparison of coupling strength between pairs from isolated ganglia and their homologs in a whole-chain preparation. The metathoracic heterogenous pairs had greater CS than their homologs from the isolated ganglion.

As in the case of the isolated ganglion preparations, and for similar reasons, the following analyses of the whole-chain preparation relate to data obtained within the frequency band 0.05–3 Hz. The SI was calculated for the 23 pairs of MNs (data provided in Supplementary Table 8). First, the seven intra-ganglion pairs were compared with their parallels in the isolated preparations and found to not significantly differ (Mann-Whitney, *p* > 0.1). This indicates that intersegmental connectivity has an insignificant or weak effect on the coordination type and consistency of intra-ganglion connections of Dep-Dep, meso- and meta-thoracic Dep-Lev, and the contiguous pairs, in the 0.05–3 Hz frequency range. Next, the 16 interganglia pairs were studied for their SI, as presented in Figure 6A. In a comparison between homolog connections in the anterior and posterior sub-networks we found significant differences only in pairs that are expected to fire in antiphase during tripod-gait locomotion (SI = 0.043 ± 0.24 and −0.25 ± 0.31 for R1Dep-R2Dep and R2Dep-R3Dep, Figure 6Ai,ii, respectively; SI = 0.353 ± 0.23 and −0.121 ± 0.29 for R1Dep-L2Lev and R2Lev-L3Dep, Figure 6Aix,xi, respectively. Mann-Whitney, *p* < 0.05). In-phase pairs were similarly synchronized in both sub-networks. Generally, as seen in the recording in Figure 6B, the anterior sub-network was active in-phase, while the posterior sub-network demonstrated tripod-like antiphase coordination, including antiphase coordination in the appropriate pairs. Moreover, the heterogenous prothoracic-metathoracic pairs: R1Dep-R3Lev and R1Dep-L3Lev showed a dysfunctional neutral coordination (SI = 0.05 ± 0.16 and 0.03 ± 0.36, Figure 6Aviii,xii, respectively), while R1Dep-R3Dep demonstrated weak in-phase coordination that approaches neutral coordination (Figures 6Aiv,B), and R1Dep-L3Dep was distinctly in-phase, unlike during tripod locomotion (Figure 6Axvi). This indicates that the prothoracic-metathoracic pathway is indirect, supporting the nearest-neighbor architecture which considers distant connections to be indirect (see Discussion for more details).

##### Coupling Strength Varies Between and Within the Sub-networks

Last, *coupling strength* (CS) between MNs in the whole-chain preparation was examined in order to study the effect of interganglia connectivity on intra-ganglion phase-lock, to enable a comparison with the isolated ganglion preparations, and also in order to reexamine and fill-in gaps in the coupling scheme previously suggested by David et al. (38). Results are presented in Figure 6C, Supplementary Figure 5, and in Supplementary Table 9. This inquiry started with the intra-ganglion pairs. Dep-Dep from different ganglia had similar CS (One-way ANOVA, *p* > 0.1, Supplementary Figure 5). In contrast, R3Dep-L3Lev and R3Dep-R3Lev were coupled significantly more strongly than R2Dep-L2Lev and R2Dep-R2Lev, respectively (*t*-test, *p* < 0.05, Figure 6Ci). A similar study of the interganglia connections followed. Another asymmetry of CS was found between reciprocal pairs (Figure 6Cii). CS of R2Dep-R3Lev was greater than that of R2Lev-R3Dep (CS = 0.28 ± 0.07 and 0.11 ± 0.06, respectively, *t*-test, *p* < 0.01) and R2Dep-L3Lev had greater CS than R2Lev-L3Dep (CS 0.22 ± 0.11 and 0.11 ± 0.06, respectively. *p* < 0.05). In addition, homolog connections in the anterior and posterior sub-networks were compared (Figure 6Ciii). Although all pairs from the posterior sub-network exhibited greater CS than their anterior homologs, the difference was significant only for the ipsilateral Dep-Lev pair (CS = 0.14 ± 0.4 and 0.28 ± 0.07 for R1Dep-R2Lev and R2Dep-R3Lev, respectively, *t*-test, *p* < 0.01). In a comparison between the posterior sub-network pairs, R2Dep-R3Lev was coupled more strongly than R2Dep-R3Dep (CS = 0.28 ± 0.07 and 0.19 ± 0.05, respectively, *t*-test, *p* < 0.05). CS was also compared between the whole-chain and the isolated preparations (Figure 6Civ, data from the isolated preparations are also presented in Figure 4C). A significant difference was found only in R3Dep-L3Lev (0.18 ± 0.05 and 0.28 ± 0.15 for isolated and whole-chain, respectively, *t*-test, *p* = 0.05), and a less significant one in R3Dep-R3Lev (0.3 ± 0.1 and 0.43 ± 0.17 for isolated and whole-chain, respectively, *t*-test, *p* = 0.069). This finding indicates that interganglion inputs to the metathoracic ganglion strengthens the coupling between depressor and levator premotor networks, while having no effect on the contralateral depressors or on the mesothoracic pairs.

## Discussion

The controversy around the origin and control of the rhythmic motor patterns for locomotion go back as far as Sherrington and Brown that have suggested an instrumental role for feedback or feedforward control, respectively (75, 76). While it is generally accepted nowadays that central pattern generating circuits are responsible for locomotion-related rhythms in practically all studied organisms from humans to insects (19, 77, 78), there are still well-established studies demonstrating, particularly for insect walking, that the currently available experimental data can be very well-explained without the need for postulating central control (79). In the current study (as in most in the field), we assume the presence of CPG circuits in the thoracic CNS that comprise conditional oscillators, i.e., require the appropriate neuromodulatory environment for producing their rhythmic output (80). Neuromodulation is known to be essential for CPGs' appropriate functioning (15, 81, 82). Here, the required modulation is provided by pilocarpine, a muscarinic agonist known to non-specifically activate premotor networks of thoracic MNs in deafferented arthropod thoracic ganglia (70, 83). Pilocarpine has been repeatedly used to induce reliable long-lasting rhythmic activity in leg-motor neurons of *P. americana* (17, 38, 71), *Manduca sexta* (66), *C. morosus* (42, 54), *S. americana* (56, 65), and *S. gregaria* (51, 52). Although pilocarpine activates both flight- and walking-CPGs, at the low concentration used here the two networks do not affect one another's output (84). In this study we analyzed the intra- and inter-ganglion motor patterns and interactions between the coxa-trochanter CPGs that control the levator-depressor networks in the American cockroach, by monitoring the pilocarpine-induced motor-patterns of levator and depressor MNs in the three isolated thoracic ganglia and in an interconnected thoracic-subesophageal ganglia chain.

### Unique Characteristics Found in Each of the Thoracic Ganglia Correspond to Their Roles in Locomotion

The cockroach's three pairs of legs differ from each other substantially in their overall size, length, foot trajectory, angle with respect to the body and to the ground, and musculature (85). The general simplified notion is that during ‘normal walking' [i.e., straight-walking on a smooth horizontal surface (86)] the front legs steer the body, are used as probers and feelers, and decelerate the body during the stance phase. The middle legs are stabilizers, pivotal axis-leg during turning, first decelerate and then accelerate the body during the stance phase, and support some of the body load. Accordingly, the hind legs are the main motor that accelerates the body forward and also support its load (50, 55, 87–89). David et al. (38) found differences in the intraganglion coordination and coupling between the meso- and meta-thoracic ganglia, as well as differences in endogenous spike frequency of depressor MNs in a semi-intact cockroach preparation. Here we examined the Levator-Depressor network in each of the three thoracic ganglia in isolation. We reveal some common features as well as differences between the ganglia, which are reflected in the nervous system connectivity and in turn affect the insect's behavior.

#### Central Control of Levator Premotor Networks

Previous studies have suggested that the levator premotor networks are predominantly controlled centrally, while those of the depressor are controlled locally (38, 45, 47, 49, 90). Our following findings support this hypothesis and suggests that interganglia coordination is best reflected in the activity of levator, rather than depressor, MNs: (i) Dep-Dep pairs showed common CS throughout the network, in both preparations, while contralateral pairs comprising a levator MN differed in their CS between ganglia. (ii) Descending inputs to the metathoracic ganglion increases the burstiness of R3Lev, as well as the CS of R3Dep-L3Lev and R3Dep-R3Lev, while not affecting R3Dep burstiness, or R3Dep-L3Dep CS. (iii) Considering that tripod gait is propagated back-to-front, and that the metathoracic ganglion dominates the overall motor pattern of a walking cockroach, our finding that in homolog and reciprocal pairs the presence of metathoracic levator accompany greater CS than when the levator is mesothoracic (Figure 6Ci,ii), also strengthens this notion. (iv) levator MNs showed similar rhythmicity between ganglia and between preparations, which indicates their oscillations are independent of local influences. In contrast, levators burstiness satisfies prothoracic > mesothoracic > metathoracic (Figure 2B), like the coherence of the contiguous pairs (i.e., Figure 3A). This suggests that burstiness is mostly influenced locally and decreases followings, or alongside, a decrease in the coherence of contiguous pairs. These findings also highlight the importance of studying levator activity.

#### Gait Transition Requires Modifications of Prothoracic and Mesothoracic Contralateral Phases

Frequency-dependent variability in coupling and inter-leg coordination have been mostly attributed to sensory-feedback and to head ganglia descending inputs (24, 79, 91–94). Our analyses, however, revealed solid evidence of endogenous frequency-dependent mechanisms in the completely isolated ganglia. Pro- and meso-thoracic homogenous pairs demonstrated frequency-dependent phase relations, indicating that gait transition is achieved also by modulating intrasegmental coordination. In addition, we found frequency-dependent coherence in the contiguous pairs. These two findings may suggest that pairs that are coordinated in antiphase during tripod locomotion, are those that are susceptible to frequency-dependent modifications. This latter finding supports Reches et al. (24), and studies of stick insect [(79), and references within], who speculated that the default coordination between all the network units is in-phase, and that functional gaits result from modifications of the default phase between some of the network units.

#### Frequency-Dependent Coherence in the Contiguous Pairs Suggests for a Mechanism of Speed-Dependent Transition Into Central Control

Next, we examined the differences in frequency-dependent variability of coherence in the contiguous pairs. Our data in Figure 3A indicate three key frequencies: one around 2 Hz, another around 5 Hz, and a third around 7 Hz, that constitute points of change in the coherence. Behavioral studies in freely-walking cockroaches found that they walk in an undefined gait at low speed, while displaying a gradual transition to tripod-gait locomotion: slow unsteady gait at ~2 steps/s, robust tripod-gait at 5 steps/s, and fast and less variable gait, which is often referred to as “running” or “trot,” above 7 step/s (27, 37, 47, 49, 95–97). Similarly, David et al. (38) reported frequency-dependent inter-leg coordination in a semi-intact preparation, gradually changing toward an ideal tripod phase, reached at 5 Hz. Interestingly, although isolated and deafferented insect preparations are known to generate much slower motor patterns than those seen in intact animals, the reported endogenous thresholds of 2, 5, and 7 Hz correspond well to the threshold for transitions into the slow, robust, and then fast tripod locomotion that was measured in intact walking cockroaches. Our results support the hypothesis that the frequency-dependent decoupling of the meso- and meta-thoracic contiguous pairs underlies a transition from the feedback to the feedforward control that enables fast locomotion in insects. During slow walking, the coupling of these contiguous pairs is strong, and local feedback governs the hemiganglion motor output. As speed increases, the general gradual decoupling allows the frequency-independent weaker central contralateral coupling to exert greater influence on the local CPG; and, if ipsilateral coupling is added, the overall central coupling prevails to dominate the motor output of the local CPG at high speed. At ~5 Hz—the estimated stride frequency limit for sensory feedback cycle-by-cycle modulation (95, 97, 98)—the local and central coupling strengths are similar, resulting in a greater central coupling influence underling the inter-leg coordination during fast locomotion. Our hypothesis is also in agreement with a report that faster cockroaches recover from perturbation within a smaller fraction of their step cycle and more uniformly than slower ones, and that they display greater uniformity in intersegmental coupling among all legs, compared to the slower cockroaches (99). Furthermore, our finding of an antero-caudal gradient in the coherence of contiguous pairs (Supplementary Figure 2) agrees with the finding of a similar gradient in stick insect (100). The strength of inter-leg coordination in the slow-walking stick insect depends on local sensory inputs to the local coxa-trochanter CPG. In the slow-walking stick insect, as oppose to our cockroach preparation, this gradient in coherence results in an antero-caudal gradient in coupling strength. Berendes et al.'s (94) finding of a speed-dependent increase in intersegmental cycle-to-cycle coupling in semi-intact walking flies is also in accord with our hypothesis. The endogenous frequency-dependent coherence of the contiguous meso- and meta-thoracic pairs also explains a fundamental characteristic of insect locomotion: the speed-dependent increase in protraction/retraction or levator/depressor duration ratios, mainly due to shortening of the stance phase duration (37, 38, 96). In a previous study we suggested a connectivity model of the levator-depressor network (38). Here we present this model with updates based on our new findings, and also provide the empirical data to support those parts of the model that were based on theoretical ideas and deductions (hereafter, “our model”; **Figure 8**). In our model we posit local hemiganglionic Lev-to-Dep inhibition and contralateral and ipsilateral Lev-to-Dep excitatory connections. Lev-to-Dep inhibition weakens toward the end of the levator burst to enable the on-time onset of the depressor burst for leg touch down. Decoupling of the contiguous pair weakens this inhibition, allowing Lev-to-Dep inter-hemiganglia excitations to induce an earlier and more intense depressor burst. This enables the same propulsion to be generated within the much shorter stance duration observed during fast running. Moreover, our results are supported by the positive correlation between burst frequency and spike frequency found in the deafferented cockroach (38).

#### The Prothoracic Network Enables Independent Activity of the Front Legs to Serve Their Unique Functioning

The cockroach front legs play a minor role in carrying the body load and in generating propulsion; however, they have a unique role in grooming, probing, steering, and negotiating obstacles. Insects turn by changing stride frequency or length without changing contralateral phases (32). Reports from various insects have shown turning also requires a change in the foot-trajectory, especially in the front legs (30, 33, 101–106). During curve walking the inner front leg performs a shorter swing, the outer counterpart extends its swing, and often also generates the perpendicular force necessary to deflect the body into the turn, while both legs retain their antiphase relation as in straight walking (102, 106), although not always (91, 103). These maneuvers require each front leg both to act independently of its counterpart leg, and to maintain an accurate coordination between its step phases and corresponding muscles. A strong coherence in the contiguous pairs enables these, first by ensuring accurate coordination of the antagonistic muscles within each leg; and second by prevailing over the central coupling that can hinder the intra-leg coordination through influence from the neighboring legs. Another mechanism that supports the front leg independence from neighboring legs is R1Dep-L1Lev weak coupling, as also found in locust (56), and the resultant endogenous neutral phase relations. Rigid contralateral Dep-Lev in-phase coordination is crucial for maintaining static stability during locomotion (2). This feature is compromised in the front legs in favor of flexible functioning. Contralateral excitation from stance- to swing-phase premotor networks have been previously suggested as centrally mediated in the cockroach (38), and sensory feedback mediated in the stick insect [rules 2 and 3 (107)], suggesting that R1Dep-L1Lev coupling is context-dependent and decoupled at need. Interestingly, coupling and decoupling of the front legs from the walking system, without compromising the coordination of the other legs, have been reported for stick insect (31). The independence of the front legs may also serve a role in negotiating obstacles, or an unexpected terrain irregularity, by adaptions of the legs' kinematics (74, 89, 108–110). Here too, strong R1Dep-R1Lev coherence, throughout the frequency range, ensures the tight intra-leg coordination that enables the front legs' unique maneuvers. Complementary to the above, the relatively strong R1Dep-L1Dep coherence ensures the contralateral legs' functional antiphase coordination even when their stepping kinematics during curve walking is far from symmetrical. This finding is in contrast to the finding of a weak R1Dep-L1Dep connection in isolated ganglion of locust, and stick insect (51, 54), perhaps due to differences between species, or quantification methods used. Moreover, the above noted findings are also in contrast with a study in stick insects (55), which have suggested that weak central coupling of R1Dep-L1Dep underlies the front legs' independent functions. Our findings of a similar CS of Dep-Dep in all isolated ganglia, alongside greater R1Dep-L1Dep coherence, and weaker R1Dep-L1Lev CS, suggest that at least in the cockroach this flexibility depends on weak Dep-Lev coupling. Last, R1Dep-L1Dep and R1Lev-L1Lev had bi-phasic coordination, as also found, but not studied further, in locust *in-vitro* preparations (51, 56), and found here to be frequency-dependent. In-phase coordination between contralateral depressor MNs was also reported for locusts (51) and stick insects (54, 111, 112). However, these *in-vitro* studies focused on burst frequencies lower than 0.5 Hz, whereas we identified a threshold for changing coordination around 2 Hz.

#### High Variability of Mesothoracic Coordination Is Crucial for Its Appropriate Locomotive Functions

Cockroach mesothoracic legs move at a range directly below the body's center of mass (113), and were found to contribute significantly more to the generation of functional and stable coordination than the other legs (31, 114, 115). During tripod locomotion, a miscalculated stance movement of the middle leg is more likely to cause a catastrophic failure than in other legs (116). Consequently, the mesothoracic hemiganglionic premotor networks must be coordinated with the neighboring hemiganglia in order to enable fast responses to perturbations and quick adaptations to immediate and unpredictable changes in velocity, direction, slope, body posture, attack angle, etc., without compromising stability. This requires a high susceptibility to modifications of the motor output. In the walking animal, a mesothoracic hemiganglion receives both central and sensory inputs from the anterior, posterior, and contralateral hemiganglia, as well as from its own local proprioceptors. These inputs modify and fine tune the motor output between and within step cycles (92). However, in the isolated ganglion these inputs are absent, and the resultant endogenous motor-output is highly variable, as can be seen in the high variability of R2Dep burstiness, and also in the transition from in-phase to erratic coordination of R2Dep-L2Dep and R2Lev-L2Lev above 2 Hz, and the practically zero synchronization index of R2Lev-L2Lev. Our data suggest that the mesothoracic intraganglion connectivity is designed for variability and susceptibility to modifications. A study on a centipede-like robot has demonstrated that straight-walking instability helps in turning maneuvers (117). This notion is also supported by the finding of weaker mesothoracic coupling in the semi-intact cockroach, and in the stick insect (38, 54), as well as the finding of bi-phasic R2Lev-L2Lev coordination in locust (56). Moreover, R2Dep-L2Dep default dysfunctional in-phase coordination, found in stick insect and locust, was suggested to be modified by sensory information to generate behaviorally relevant coordination (24, 54). Overall, these findings indicate that insects share similar principles of mesothoracic intraganglion connectivity, and that their locomotion behavior may be different due to the application of different effectors (e.g., neuromodulators, sensory inputs, etc.) on a similar default neuronal infrastructure.

#### The Metathoracic Network Presents Consistent Tripod-Like Coordination

The hind legs are the main motor that propel the body forward (1), and support much of the body load (88). The metathoracic ganglion that controls the hind legs receives ascending inputs from the abdominal ganglia and the cerci, including direct inputs from the giant interneurons that mediate the cockroach escape response (118). The isolated metathoracic network presents the consistent tripod-like coordination that is expected from the main motor during forward locomotion. R3Dep-L3Dep and R3Lev-L3Lev persistent antiphase coordination suggests the existence of a unique metathoracic central and frequency-independent contralateral mutual inhibition mechanism, which also explains the greater CS in comparison to the other ganglia, and prevents co-swinging of the hind legs. Additional evidence of such a mechanism is provided by the relatively high coherence of R3Dep-L3Lev throughout most of the frequency range, which alongside the consistent in-phase coordination is crucial for static stability of the hind legs and, therefore, the whole-body (2). The findings of a consistent antiphase coordination of R3Dep-L3Dep and R3Lev-L3Lev in locust isolated ganglion, and of a stronger coupling of the metathoracic Dep-Lev in comparison to the other ganglia (51, 56, 65), alongside the contralateral application of Cruse's rules II and III (107, 119) and the finding of a tendency to antiphase coordination of R3Dep-L3Dep in stick insects (54), suggest that this feature is conserved at least in hemimetabola insects. The extreme lower coherence of R3Dep-R3Lev suggests that local influences and accurate intra-hemiganglion coordination is less important in the hind legs. This notion is supported by our findings of low and more variable levator burstiness than in the other ganglia, which indicate that in the hind legs the accuracy of stepping is compromised in order to enable the high frequency leg cycling necessary for cockroach fast locomotion (1).

### The Whole-Chain Preparation

Previous experimental research of deafferented locusts and stick insects focused solely on depressor MNs and found that all six of them are synchronized in-phase (51, 54), as also suggested in a recent modeling study (24). These and other experimental studies of deafferented stick insects, and crustaceans have found only in-phase coordination between ipsilateral homolog MNs (42, 120–122). Our current cockroach preparation motor-patterns were found to profoundly differ from the above-noted findings.

#### The Effects of Intersegmental Connectivity on the Intrasegmental Motor Patterns

By comparing between the same pairs of MNs in the isolated and the whole-chain preparations, we examined the effect of the centrally generated inter-ganglia inputs on the intra-ganglionic motor outputs. Our finding of lower rhythmicity of depressor MNs in the whole-chain preparation, indicates that they are more susceptible than levator MNs to intersegmental interferences. This agrees well with the model we present in **Figure 8**, in which both levator and depressor premotor INs receive input from an oscillator that in turn is influenced by the neighboring oscillators. The depressors, however, also receive direct inputs from levator premotor networks in the neighboring ganglia, which increases their motor pattern variability in comparison to that in the isolated preparations and to the levator networks. For example, although mesothoracic Dep-Dep had a similar mean phase in both our preparations, the phase was 3-fold less variable in the whole-chain preparation. R1Dep-L1Dep displayed different coordination in the two preparations: frequency-dependent bi-phasic coordination in the isolated preparation; and a consistent in-phase coordination in the presence of intersegmental inputs. Descending inputs from the SEG have been found to induce in-phase synchronization between contralateral depressors in a locust *in-vitro* preparation, with a stronger effect on the prothoracic pair (52). The prothoracic MNs' low variable rhythmicity can suggest that this mechanism is common to locusts and cockroaches, although the R3Dep-L3Dep antiphase coordination could indicate that in the cockroach the metathoracic motor output is less influenced by the SEG descending inputs than in the locust. An even greater effect of intersegmental connectivity was that of stabilizing the motor pattern of the metathoracic MNs by increasing the CS of heterogeneous pairs and the levator MNs burstiness. In contrast, R3Dep-L3Dep was unaffected by descending inputs. These findings indicate that levator premotor networks are the targets of intersegmental influence on the cockroach metathoracic ganglion. In stick insects, mesothoracic inputs were found to be necessary for regular stepping of the metathoracic legs (31), as well as in strengthening intrasegmental coupling in intact and isolated deafferented preparations (55, 100). Moreover, mesothoracic-metathoracic connectivity was found to increase R2Dep-L2Dep coupling in stick insects (55), and decrease R2Dep-L2Dep phase variability in our cockroach preparation (Supplementary Figure 3). More generally, with the exception of R1Dep-L1Dep, intersegmental connectivity did not affect synchronization of the pairs investigated here, indicating that gait modifications are mostly executed by altering the coordination between, and not within, the ganglionic networks.

#### The Anterior Sub-network Transitions Into Tripod-Appropriate Coordination While the Posterior Sub-Network Presents Tripod-Appropriate Coordination Throughout the Frequency Range

During ‘normal walking' cockroaches have presented similar phases of prothoracic-mesothoracic and mesothoracic-metathoracic legs, e.g., R1–R2 and R2–R3 present a similar mean phase (25, 37, 123). In contrast, our preparation exhibited significant asymmetries between the phases of homolog interganglia pairs. To investigate this, we divided the network into anterior and posterior sub-networks (prothoracic-mesothoracic and mesothoracic-metathoracic, respectively, Figure 5B). The anterior sub-network's coordination transitioned into tripod gait phases at 2 Hz (Figure 5Ci,ix), along the beginning of a sharp change in coherence of R1Dep-R1Lev and R2Dep-R2Lev. Considering R1Dep-L1Dep and R1Lev-L1Lev phase inversion above 2–3 Hz (Figure 3Bi,ii, respectively), these findings indicate that the prothoracic ganglion dominates this sub-network at low frequencies. In the posterior sub-network, this transition occurred at 5 Hz, with one exception - R2Dep-R3Lev maintained a consistent in-phase coordination, unlike its reciprocal pair R2Lev-R3Dep (Figure 5Cvi,vii). The frequency-dependent transition of R2Lev-R3Dep into antiphase coordination could indicate that Lev-to-Dep ipsilateral excitation (38) is overridden at high frequencies, which results in antiphase activity. The finding of weaker descending than ascending mesothoracic-to-metathoracic coupling in *P. americana* (38, 99) supports this notion. In the heterogenous diagonal pairs, stronger coupling accompanied the dysfunctional in-phase coordination of R2Dep-L3Lev, while weaker coupling in R1Dep-L2Lev accompanied the bi-phasic coordination, and the weakest coupling, of R2Lev-L3Dep, accompanied a consistent tripod-appropriate antiphase coordination. Overall, we conclude that Dep-Lev pairs which are coordinated in-phase during tripod locomotion depend on a stronger CS to generate tripod coordination while pairs that are antiphase coordinated during tripod locomotion depend on a weak CS to generate appropriate coordination.

#### Ipsilateral Coordination Overturn at 5 Hz Suggests for an Endogenous Coordination Which Comprises Simultaneous Aerial Phases of the Ipsilateral Middle and Hind Legs

Three out of four ipsilateral pairs in the posterior sub-network inverted their coordination from tripod-appropriate asymmetry into a different motor-pattern around 5 Hz, which corresponds to the frequency threshold for the transition from local feedback-dominated control into central feedforward-dominated control. This new distinctive state corresponds to overlapping aerial phases of ipsilateral legs in an intact running cockroach, as also found between R2Lev and R3Lev in 40% of the burst-cycles in semi-intact cockroaches (38). Simultaneous swing phases of contralateral legs were reported for insects using the uncommon gallop, quadrupedal, or bipedal gaits (124, 125), and a faster-than-tripod gait has been characterized in the cockroach *N. cinereal* (123). However, we are unaware of evidence in the literature for ipsilateral mesothoracic-metathoracic synchronized swing movements in intact walking insects. Following Weihmann et al.'s (123) definition that tripod gait satisfies 282° ≤ Φ ≤ 72° between the front and hind ipsilateral legs, our findings indicate that *P. american*a still satisfies tripod coordination also at frequencies > 5 Hz. The differences in biomechanics between *P. americana* and other insects (1, 37, 39), with the underlying neural mechanism depicted here, may enable *P. americana* to maintain tripod coordination and its benefits throughout its speed range, by altering the ipsilateral coordination to include aerial phases without altering the contralateral, diagonal and even pro-to-meta thoracic phase relations. Slow and fast tripod gaits have been previously distinguished in cockroaches (27, 96), and a change from relying on the static stability of the tripod footfall pattern to a dynamic stability during very fast running was reported previously (2) and further support this notion.

#### The Mesothoracic Ganglion Serves as a Subordinate Mediator Between the Dominant Pro- and Meta-Thoracic Ganglia

Unlike the three other prothoracic-metathoracic pairs, R1Dep-R3Dep showed a consistent tripod-appropriate phase. The current lack of evidence for direct connectivity between the prothoracic and metathoracic motor networks suggests that this stable functional phase may be coordinated through the mesothoracic ganglion. One way of achieving such coordination is through a consistent in-phase coordination of diagonal Lev-Lev and Dep-Dep pairs, as found here (Figure 5Cxiii,xiv,xv). Furthermore, R1Dep-L3Dep, R1Dep-L3Lev, and R1Dep-R3Lev showed a dysfunctional motor-pattern that is likely to have resulted from the simultaneous activity of two different networks with shared elements, rather than from the coupling between distant parts of a single network. Hence, we suggest that the anterior and posterior sub-networks are separate networks that are connected and functionally coupled via a shared element- the mesothoracic network- to form the thoracic locomotion control network. The demonstrated ability of functionally specialized legs to couple to, or decouple from, the other legs, supports this notion (31, 74, 126). Moreover, although each ganglion can dominate the overall behavior in different contexts (51), our data suggests that the prothoracic ganglion dominates the overall motor pattern at frequency <2 Hz, the metathoracic ganglion dominates during faster locomotion, and the mesothoracic ganglion mostly serve as a subordinate mediator connecting the sub-networks and following the motor pattern of the current dominant ganglion. This notion is supported by the relatively weak coupling found in R2Dep-R2Lev, and R2Dep-L2Lev (Figure 6Ci), which renders the mesothoracic network components more susceptible to influences from neighboring ganglia, since weak coupling is more easily overridden. R2Dep-R2Lev weaker coupling in the whole-chain preparation in comparison to the isolated preparation (Figure 6Civ) provides additional support to this notion, as well as the mixed prothoracic and metathoracic characteristics presented by the isolated mesothoracic ganglion. Last, in the posterior sub-network, interganglion Dep-Lev had a weaker coupling if the levator was mesothoracic than if it was metathoracic (Figure 6Cii). David et al. (38) reported that meso-metathoracic descending coupling is weaker than the parallel ascending coupling. These facts suggests that Dep-Lev interganglia coupling between ganglia depends on the levator premotor networks. Weaker mesothoracic levator's coupling, and its resultant more variable phases, further indicate for the mesothoracic function as a subordinate mediator.

#### Rules for Couplings Between Cockroach Levator-Depressor Motor Centers

**Figure 7:**
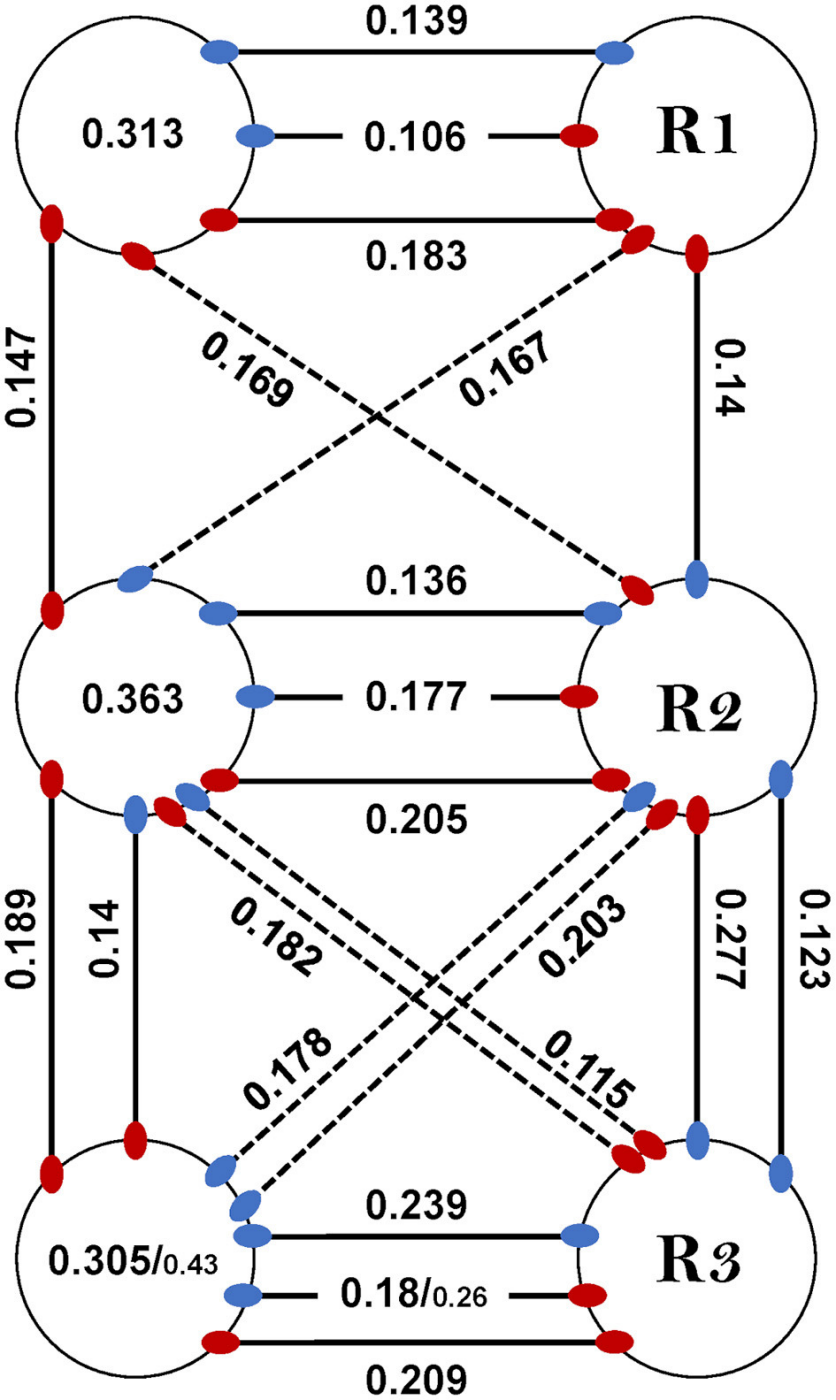
Coupling scheme. Intraganglion and interganglia values of CS were obtained from the isolated and whole-chain preparations, respectively. Where a significant difference between the preparations was found we present two values as isolated/whole-chain. Following a nearest-neighbor architecture, the diagonal connections (dashed lines) are considered functional and not direct, and the prothoracic-metathoracic connections are absent (see text). Red or blue indicate for depressor or levator efferent, respectively (e.g., red-blue connection represents a depressor-levator connection). Values of contiguous pairs are presented within the corresponding circle. R1, R2, and R3 indicate for the right prothoracic, mesothoracic, and metathoracic ganglion, respectively. Pairs from the posterior sub-network present greater coupling than their homolog pairs in the anterior sub-network. Interganglia connectivity increases intraganglion coupling in the metathorax and decreases it in the mesothorax. Intraganglion Dep-Dep coupling is unaffected by interganglia connectivity.

**Figure 8:**
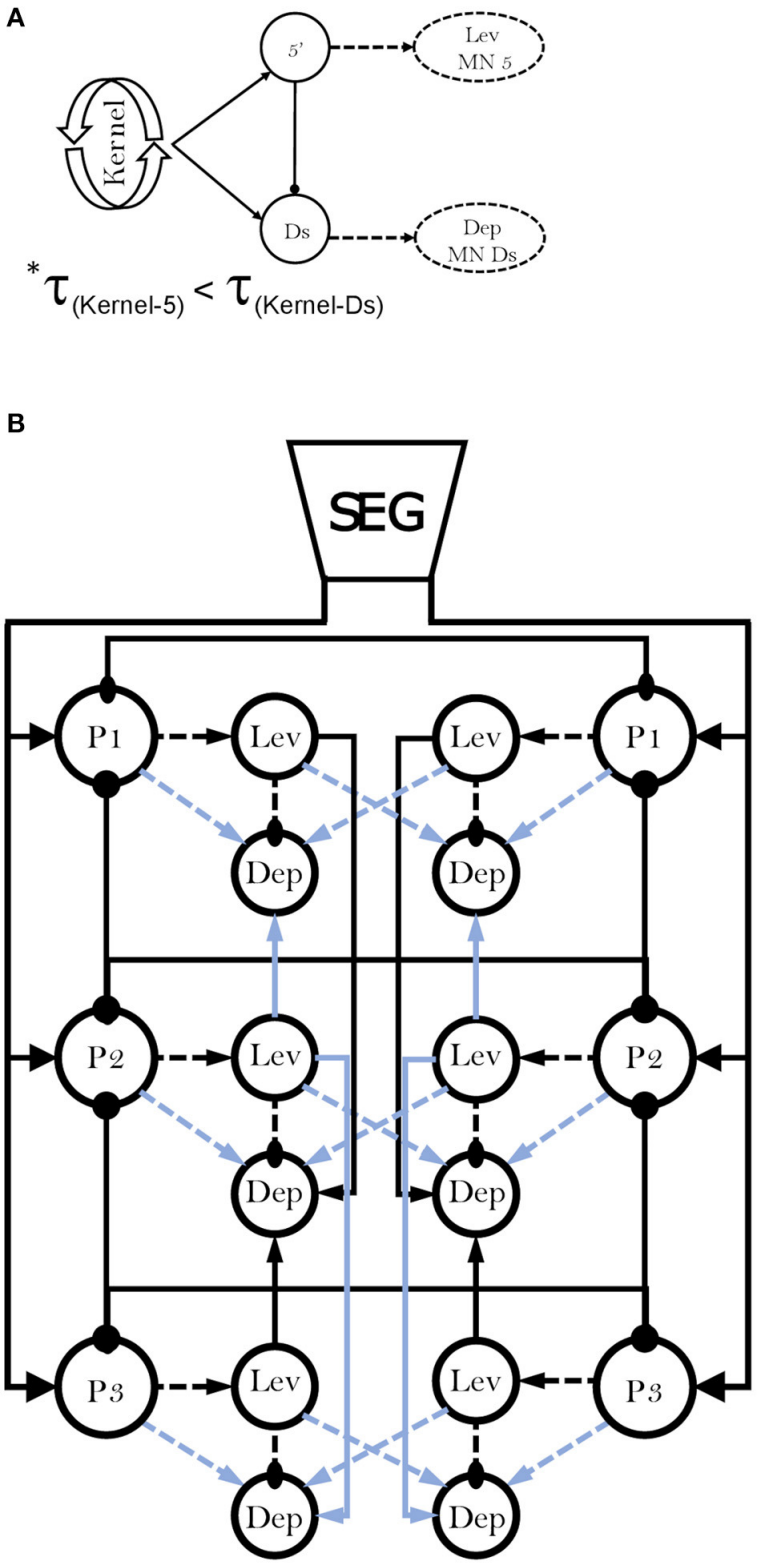
Parsimonious connectivity model [modified from (38)]. Circles and arrows indicate for inhibitory and excitatory connections, respectively. (**A)** Reduced representation of the hemiganglionic CPG. 5' and Ds are levator and depressor interneuron pools that innervate the slow depressor MN (Ds) and levator MN 5'. ^*^correction of a typo in Figure 7A in David et al. (38), in which the smaller-than sign (<) is mistakenly presented as larger-than (>). (**B)** Solid line- between CPGs, dashed line- within CPG. Black and blue represent strong and weak connections, respectively. P1, P2, and P3 indicates for pro-, meso-, and meta-thoracic hemiganglionic pacemaker. The model incorporates a descending excitatory drive from the SEG to the thoracic ganglia oscillators, alongside mutual inhibition between CPGs and direct excitation of the depressor interneuron pool by neighboring levators interneuron pools. In addition, mesothoracic inputs are stronger than mesothoracic outputs, and levator interneuron pools receives a single excitatory input, while the depressor interneuron pools are innervated simultaneously by three excitatory inputs and one inhibitory input.

Finally, we used our data to posit a new coupling scheme (Figure 7), which updates and fills in gaps in the coupling scheme published by David et al. (38), and the coupling rules it offered. For intraganglion connections data were obtained from our isolated preparations and for intersegmental connections data were obtained from our whole-chain preparation. This approach is supported by our finding of significant influence of interganglion connectivity on intraganglion couplings only for R3Dep-R3Lev and R3Dep-L3Lev (Figure 6Civ), for which we denote both isolated and whole-chain couplings. Our scheme is restricted due to a lack of sufficient data on the intraganglion Lev-Lev pairs in the whole-chain preparation. The identified coupling rules also manifest in our connectivity model (Figure 8) which was thoroughly discussed in David et al. (38), and will be discussed here only in light of the new findings which modifies it. Therefore, *Rule 1* “levator INs excite neighboring depressor INs” and *Rule 6* “meta-meso ascending coupling is stronger than meso-meta descending coupling” are not discussed here. Our recent findings (Figure 7) disagree with *Rule 2* “Ipsilateral connections are coupled stronger than contralateral ones.” Dep-Dep pairs which were not investigated by David et al. (38), were found here to contradict this rule, as did Lev-Lev and Dep-Lev pairs. We attribute the difference between our current and previous findings to ascending sensory inputs from the abdominal ganglia, which were the only inputs that were not deafferented in David et al. (38). This also indicates that abdominal sensory signals suffice to increase the ipsilateral coupling strength, at least in the posterior sub-network. In contrast, *Rule 3* “Lev-Dep is stronger than the parallel Lev-Lev” is supported by our new findings (Figure 7), except for R1Dep-L1Lev. We further compared these couplings to those of the parallel Dep-Dep pairs and found no consistent difference, and that Dep-Dep pairs were generally similar in their coupling strength. Our findings also support *Rule 4* “Metathoracic coupling is stronger than mesothoracic coupling” for Lev-Lev and Dep-Lev pairs, as in the semi-intact cockroach, but not for Dep-Dep pairs. We therefore redefine *Rule 4* as: “Pairs comprising a metathoracic levator are coupled more strongly than homolog pairs comprising mesothoracic levators.” To this we add our findings from the whole-chain preparation and note that pairs in the posterior sub-network are generally coupled more strongly than their homolog pairs in the anterior sub-network. *Rule 5* “Diagonal coupling is functional and not direct.” This assumption is derived from the nearest-neighbor architecture inferred from Spirito and Mushrush (96), and supported by Couzin-Fuchs et al. (99) and Aminzare et al. (127), and by findings from crustaceans swimming control network (128, 129). Diagonal intersegmental pathways were identified in the cockroach and locust (52, 130–132), but were described as mediating sensory information or brain commands. Our findings of highly variable and dysfunctional phases between prothoracic and metathoracic MNs, in addition to the extremely weak CS of R1Dep-L3Dep, as also predicted by a simulation study in stick insects (79), all support this architecture at least for these long-distance connections. We note, however, that a different modeling effort of the stick insect locomotion control network postulated a direct coupling between the prothoracic and metathoracic ganglia (133).

## Data Availability Statement

The original contributions presented in the study are included in the article/Supplementary Material, further inquiries can be directed to the corresponding author/s.

## Author Contributions

ID and AA designed the study and wrote the manuscript. ID conducted the experiments and analyzed the data. Both authors contributed to the article and approved the submitted version.

## Conflict of Interest

The authors declare that the research was conducted in the absence of any commercial or financial relationships that could be construed as a potential conflict of interest.
