# Supplementary material for: From Motor-Output to Connectivity: An In-Depth Study of in-vitro Rhythmic Patterns in the Cockroach Periplaneta americana
Source: Front Insect Sci. 2021 May 20;1:655933. doi: 10.3389/finsc.2021.655933 (PMC10926548; doi:10.3389/finsc.2021.655933)
Supplement: Supplementary file 1 [file Data_Sheet_1.PDF]

Supplementary Materials

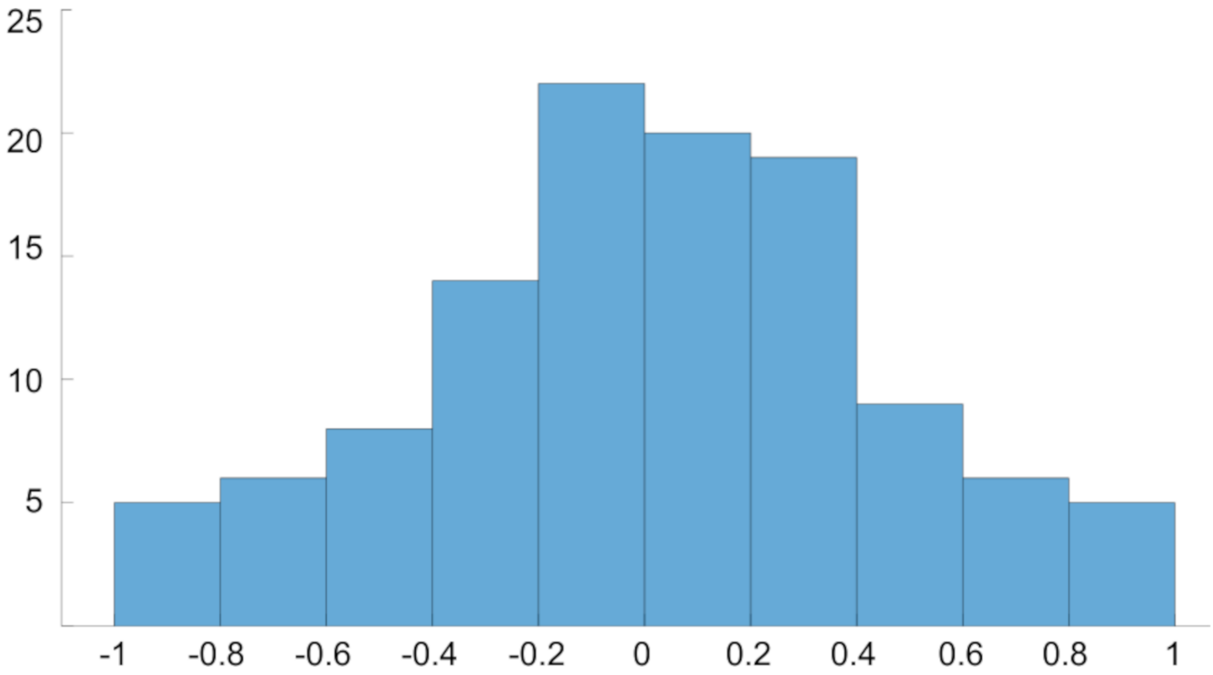

Figure S1. Calculated confidence intervals for synchronization index. Confidence intervals (CI) of ±0.081 SI (±5%) were calculated for a group of 113 SI values randomly sampled from a list of 1,130 values calculated for pairs of MNs in which each MN was sampled from a different experiment and, therefore, should have no coupling with its counterpart. The figure presents the distribution of the random sampled SI values. The mean was found to not significantly differ from zero (two-sided t-test against hypothetical mean=0, p=0.72). Confidence intervals for SI were calculated for this sample.

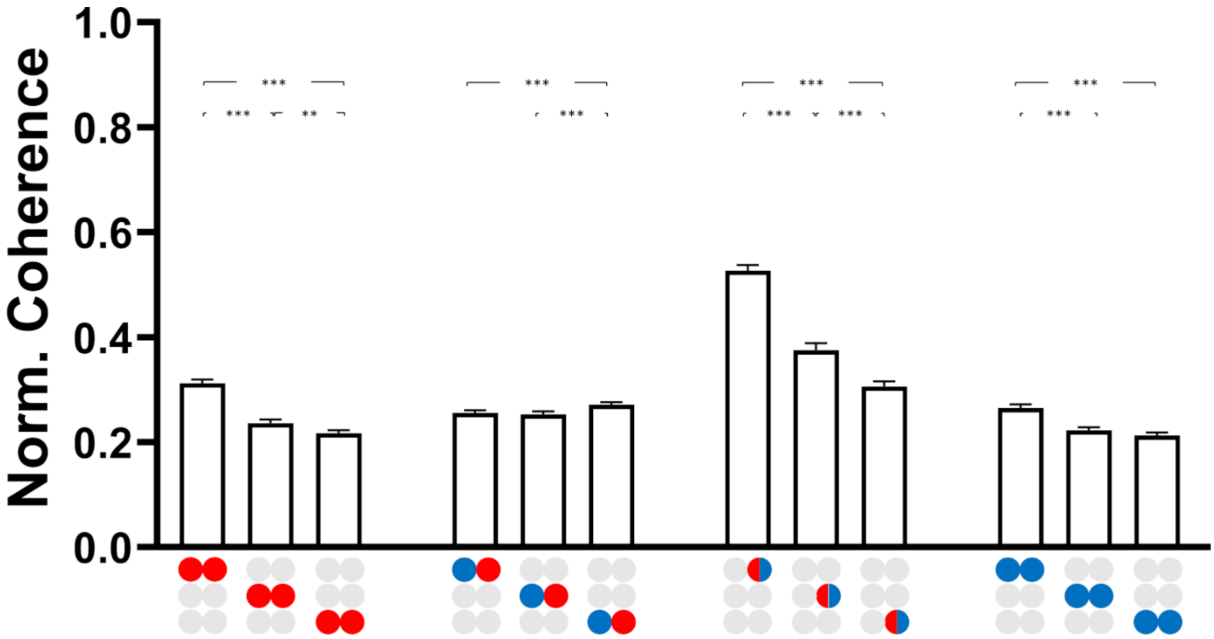

Figure S2. Coherence in isolated ganglia preparations. Red and blue denote depressor and levator nerves, respectively. Mixed colors denote a pair of levator and depressor within a hemiganglion (contiguous). ** & ***denote for $p<0.01$ & 0.001. Coherence values were normalized with the greatest value calculated in this work and are presented as Mean+CI.

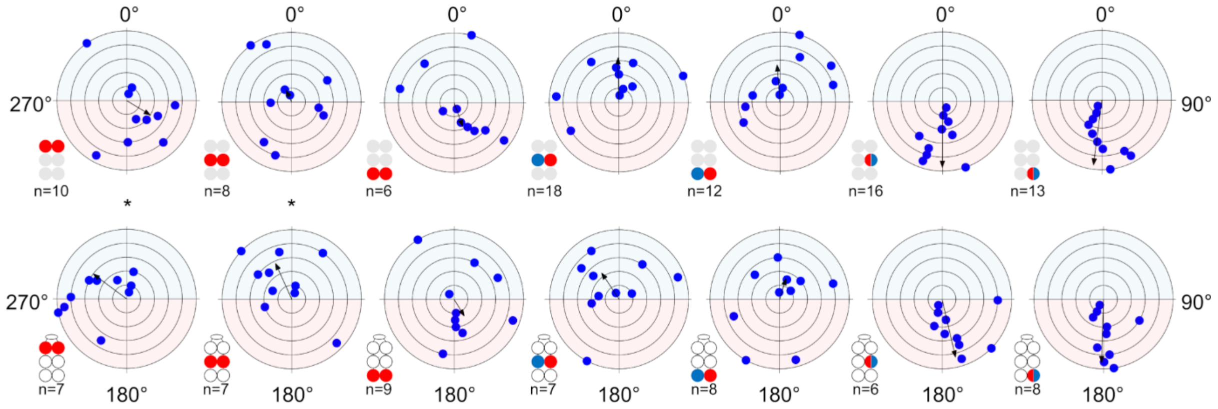

Figure S3. **A comparison of intraganglion connections between isolated and whole-chain preparations.** The circular-linear plots are colored pale blue (270° → 90°) and red (90° → 270°) to represent in-phase and antiphase coordination, respectively. The illustrations are colored according to the motor nerves function - red and blue for depressor and levator, respectively. Mixed colors denote a pair of levator and depressor within a hemiganglion. Illustrations with gray circles represent the isolated preparation (top panel) and illustrations with black empty circles represent the whole-chain preparation (bottom panel). * denotes p<0.05 (Watson-Williams's test). Each point in the plots represents the mean phase of a 1 Hz bin. Grid lines = 2Hz. The black arrow is the vector of phase, calculated for the entire 10 Hz range of frequencies. Only R1Dep-L1Dep present a significant difference in coordination between the preparations.

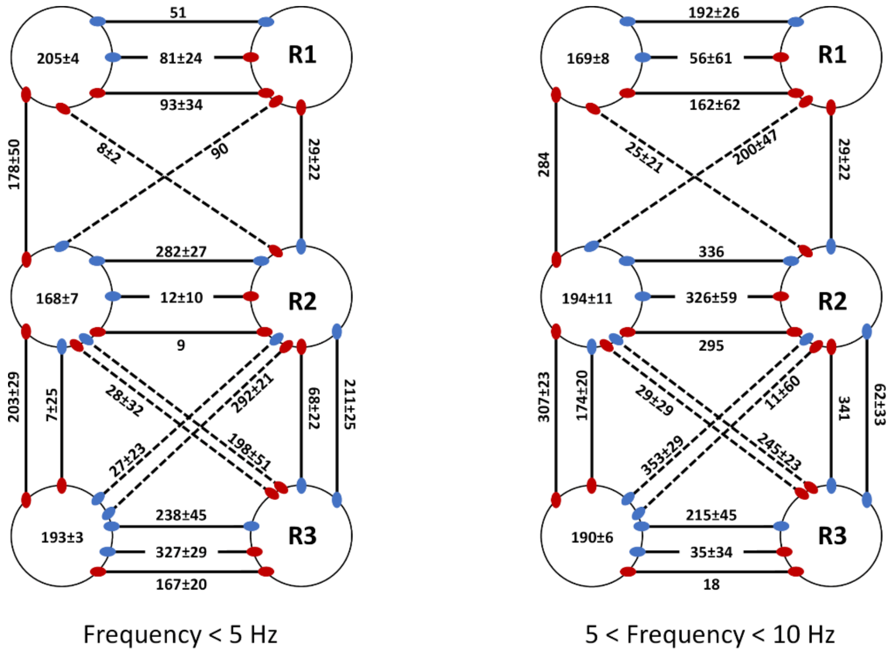

Figure S4. **Frequency-dependent phases.** Schemes of the phase relations at low- and high-frequency (left and right, respectively) activity. Data are presented as mean phase±SE (when circular SE could be calculated from the data). Intraganglion and interganglia phases were obtained from the isolated and whole-chain preparations, respectively. Red or blue indicate for depressor or levator efferent, respectively (e.g., red-blue connection represents a depressor-levator connection). Values of contiguous pairs are presented within the corresponding circle. R1, R2 & R3 indicate for the right prothoracic, mesothoracic, and metathoracic ganglion, respectively.

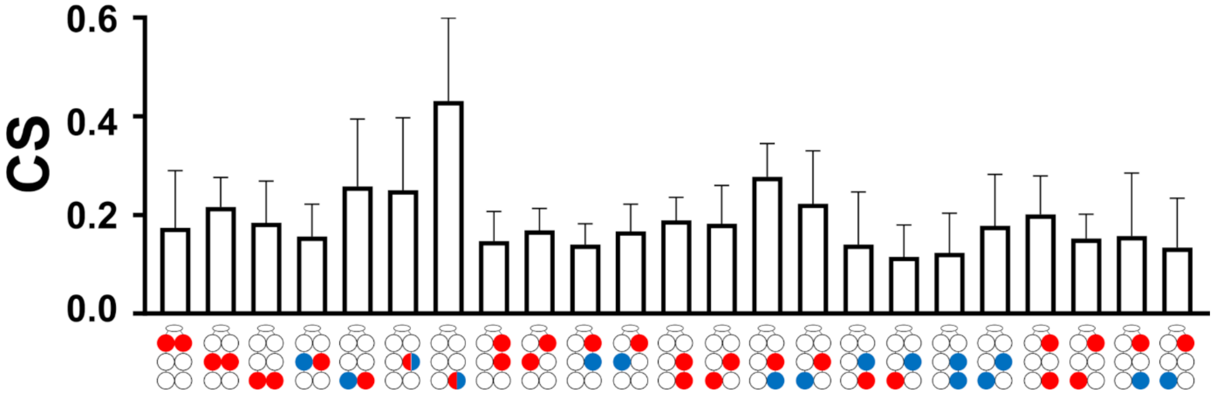

Figure S5. **Coupling strength in the whole-chain preparation.** Data are presented as mean+CI. Illustrations are colored according to the motor nerves function - red and blue for depressor and levator, respectively. Mixed colors denote a pair of levator and depressor within a hemiganglion.

Burst.	R1D-i	R1D-w	R2D-i	R2D-w	R3D-i	R3D-w	R1L-i	R1L-w	R2L-i	R2L-w	R3L-i	R3L-w
25%	0.127	0.043	-0.307	0.046	0.053	-0.004	0.362	0.396	0.205	0.106	-0.141	0.337
Median	0.276	0.338	0.157	0.270	0.248	0.279	0.592	0.670	0.450	0.350	0.286	0.588
75%	0.523	0.436	0.327	0.436	0.459	0.428	0.720	0.823	0.628	0.507	0.629	0.637
Mean	0.275	0.275	0.074	0.221	0.175	0.189	0.540	0.625	0.413	0.332	0.253	0.511
SD	0.290	0.293	0.469	0.341	0.373	0.303	0.237	0.232	0.268	0.225	0.364	0.179
±CI	0.136	0.124	0.198	0.144	0.186	0.125	0.122	0.243	0.106	0.161	0.181	0.128
n units	20	24	24	24	18	25	17	6	27	10	18	10
N animals	11	17	15	17	10	16	11	5	15	8	10	9

Table S1. **Burstiness of levator and depressor nerves in isolated and whole-chain preparations.** i and w indicate for isolated and whole-chain preparations. N animals and n units denote the number of experiments and the number of pairs of the noted type. A recording can also comprise two similar pairs, e.g., R2Dep-L2Lev and L2Dep-R2Lev.

Rhythm	R1D-i	R1D-w	R2D-i	R2D-w	R3D-i	R3D-w	R1L-i	R1L-w	R2L-i	R2L-w	R3L-i	R3L-w
25%	0.080	0.054	0.196	0.080	0.154	0.069	0.125	0.085	0.085	0.041	0.084	0.057
Median	0.209	0.101	0.443	0.133	0.197	0.152	0.191	0.121	0.188	0.168	0.219	0.078
75%	0.426	0.233	0.674	0.253	0.493	0.261	0.254	0.186	0.470	0.316	0.315	0.266
Mean	0.307	0.155	0.429	0.204	0.326	0.230	0.230	0.136	0.280	0.187	0.224	0.163
SD	0.272	0.134	0.269	0.187	0.262	0.238	0.158	0.087	0.215	0.148	0.164	0.159
±CI	0.092	0.056	0.114	0.079	0.130	0.098	0.081	0.092	0.085	0.106	0.082	0.114
n units	20	24	24	24	18	25	17	6	27	10	18	10
N animals	11	17	15	17	10	16	11	5	15	8	10	9

Table S2. **Rhythmicity of levator and depressor nerves in isolated and whole-chain preparations.**

Norm. Coherence		Mean	SD	SE	±95% CI	n units	N animals
Pro	R1D-L1D	0.314	0.082	0.003	0.007	10	10
	R1D-L1L	0.256	0.062	0.003	0.005	14	10
	R1D-R1L	0.528	0.134	0.005	0.011	14	10
	R1L-L1L	0.265	0.090	0.004	0.007	8	8
Meso	R2D-L2D	0.237	0.088	0.004	0.007	8	8
	R2D-L2L	0.254	0.063	0.003	0.005	18	13
	R2D-R2L	0.376	0.167	0.007	0.014	16	11
	R2L-L2L	0.223	0.069	0.003	0.006	10	10
Meta	R3D-L3D	0.218	0.069	0.003	0.006	8	6
	R3D-L3L	0.272	0.064	0.003	0.005	16	8
	R3D-R3L	0.307	0.123	0.005	0.010	17	9
	R3L-L3L	0.213	0.067	0.003	0.005	8	6

Table S3. **Normalized coherence of phase between motor nerves in the isolated ganglia preparations.** Coherence was normalized for the highest values measured in this work for the analyzed range of frequencies.

Hz	R1D-L1D	R1D-L1L	R1D-R1L	R1L-L1L	R2D-L2D	R2D-L2L	R2D-R2L	R2L-L2L	R3D-L3D	R3D-L3L	R3D-R3L	R3L-L3L
0.05-1	18	151	214	27	347	7	150	348	157	358	192	181
1-2	23	102	202	69	332	19	178	283	233	11	194	250
2-3	153	53	205	358	268	41	164	266	161	349	186	118
3-4	134	89	208	187	102	0	182	295	151	283	194	291
4-5	116	7	196	219	113	356	164	218	144	262	201	283
5-6	178	331	186	226	59	20	214	315	131	240	181	210
6-7	96	62	179	243	214	326	197	172	323	24	188	17
7-8	139	348	156	149	197	239	197	146	284	73	181	216
8-9	209	145	152	212	337	275	199	15	127	55	194	157
9-10	325	112	173	107	324	68	161	326	15	16	205	253
Mean	121	72	187	181	335	359	181	290	160	356	192	227
Vector	0.40	0.53	0.94	0.27	0.19	0.64	0.94	0.43	0.36	0.53	0.99	0.46
SD	78	64	21	93	105	54	20	75	82	65	7	71
N	10	10	10	8	8	13	11	10	6	8	9	6

Table S4. **Phase relations between motor nerves binned by frequency.** Values of phase are rounded to save space. Each bin comprised 60 frequencies, except the first bin, which comprised 58 (the two first frequencies are omitted due to presenting an artifact).

SI (Isolated)		Mean	SD	SE	±95% CI	n units	N animals
Pro	R1D-L1D	0.125	0.510	0.161	0.365	10	10
	R1D-L1L	-0.062	0.180	0.048	0.104	14	10
	R1D-R1L	-0.542	0.420	0.112	0.242	14	10
	R1L-L1L	0.122	0.356	0.126	0.298	8	8
Meso	R2D-L2D	0.097	0.582	0.206	0.486	8	8
	R2D-L2L	0.355	0.315	0.074	0.157	18	13
	R2D-R2L	-0.294	0.698	0.175	0.372	16	11
	R2L-L2L	0.046	0.346	0.110	0.248	10	10
Meta	R3D-L3D	-0.483	0.329	0.134	0.345	6	6
	R3D-L3L	0.396	0.328	0.095	0.209	12	8
	R3D-R3L	-0.488	0.525	0.146	0.317	13	9
	R3L-L3L	-0.418	0.365	0.149	0.382	6	6

Table S5. **Synchronization index in the isolated ganglia preparations.**

CS (Isolated)		Mean	SD	SE	±95% CI	n units	N animals
Pro	R1D-L1D	0.183	0.116	0.037	0.083	10	10
	R1D-L1L	0.106	0.047	0.013	0.027	14	10
	R1D-R1L	0.313	0.131	0.035	0.076	14	10
	R1L-L1L	0.139	0.059	0.021	0.049	8	8
Meso	R2D-L2D	0.205	0.103	0.036	0.086	8	8
	R2D-L2L	0.177	0.123	0.029	0.061	18	13
	R2D-R2L	0.363	0.184	0.046	0.098	16	11
	R2L-L2L	0.136	0.091	0.029	0.065	10	10
Meta	R3D-L3D	0.180	0.105	0.043	0.110	6	6
	R3D-L3L	0.180	0.084	0.024	0.054	12	8
	R3D-R3L	0.305	0.169	0.047	0.102	13	9
	R3L-L3L	0.239	0.117	0.048	0.123	6	6

Table S6. **Coupling strength between pairs of motor nerves in the isolated ganglion.**

Hz	0.05-1	1-2	2-3	3-4	4-5	5-6	6-7	7-8	8-9	9-10	Mean	Vector	SD	N
R1D-L1D	19	19	334	14	302	296	212	272	263	259	307	0.60	58	7
R2D-L2D	26	16	294	254	320	306	345	34	134	314	336	0.56	61	7
R3D-L3D	317	172	177	176	166	29	64	191	110	329	149	0.28	92	7
R2D-L2L	333	66	279	261	312	34	309	330	69	208	326	0.45	72	6
R3D-L3L	356	53	21	49	315	358	250	74	165	209	16	0.31	88	9
R2D-R2L	211	197	170	196	176	159	159	91	162	135	167	0.85	32	8
R3D-R3L	213	208	210	173	175	121	187	174	180	171	182	0.91	25	8
R1D-R2D	295	72	192	175	171	249	275	56	139	318	207	0.22	100	13
R1D-R2L	22	83	43	337	20	8	347	76	250	36	22	0.68	50	16
R1D-L2D	15	5	9	4	9	7	20	343	78	41	16	0.92	24	5
R1D-L2L	354	344	100	142	175	349	175	135	225	223	166	0.22	99	6
R2D-R3D	231	144	153	245	232	244	322	289	31	304	253	0.44	74	9
R2D-R3L	70	91	74	358	95	353	78	274	335	171	52	0.44	73	9
R2L-R3D	91	42	350	338	312	158	215	202	166	128	139	0.13	116	7
R2D-L3D	158	24	354	352	55	48	325	325	98	60	28	0.59	59	6
R2D-L3L	351	279	288	296	253	266	89	15	64	311	315	0.51	67	5
R2L-L3D	161	215	238	348	141	267	270	300	174	204	227	0.52	65	7
R2L-R3L	200	153	261	170	278	84	64	147	38	325	153	0.17	108	5
R2L-L3L	46	15	320	67	37	5	349	41	263	359	11	0.74	45	5
R1D-R3D	47	187	351	291	35	8	337	89	311	22	5	0.54	64	9
R1D-R3L	23	241	243	22	59	137	324	44	288	214	333	0.19	104	11
R1D-L3D	329	315	353	311	5	117	113	179	261	252	312	0.29	91	5
R1D-L3L	130	138	303	152	97	87	37	157	126	111	116	0.65	54	7

Table S7. **Frequency-dependent phases in the whole-chain preparation.** Values of phase are rounded to save space. Each bin comprised 60 frequencies, except the first bin, which comprised 58 (the two first frequencies are omitted due to presenting an artifact).

SI W-C	25%	Median	75%	Mean	SD	SE	±95% CI	n units	N animals
R1D-L1D	-0.078	0.085	0.718	0.261	0.415	0.157	0.384	7	7
R2D-L2D	-0.020	0.295	0.510	0.207	0.442	0.167	0.408	7	7
R2D-L2L	0.078	0.231	0.393	0.185	0.325	0.123	0.300	7	7
R2D-R2L	-0.884	-0.699	-0.168	-0.572	0.369	0.151	0.387	6	6
R3D-L3D	-0.682	-0.436	-0.076	-0.410	0.316	0.105	0.243	9	9
R3D-L3L	0.151	0.360	0.751	0.315	0.579	0.205	0.484	8	8
R3D-R3L	-0.942	-0.705	0.606	-0.312	0.779	0.276	0.652	8	8
R1D-R2D	-0.204	0.099	0.313	0.043	0.395	0.110	0.239	13	13
R1D-L2D	0.058	0.421	0.600	0.342	0.312	0.078	0.166	16	16
R1D-R2L	0.208	0.316	0.380	0.298	0.115	0.052	0.143	5	5
R1D-L2L	0.161	0.371	0.537	0.353	0.220	0.090	0.231	6	6
R2D-R3D	-0.654	-0.296	0.015	-0.250	0.440	0.139	0.312	10	9
R2D-L3D	-0.044	0.262	0.655	0.266	0.392	0.124	0.280	10	9
R2D-R3L	-0.335	-0.050	0.750	0.158	0.597	0.226	0.552	7	7
R2D-L3L	-0.247	0.073	0.701	0.177	0.476	0.194	0.500	6	6
R2L-R3D	-0.117	0.121	0.440	0.153	0.334	0.150	0.415	5	5
R2L-L3D	-0.427	-0.130	0.102	-0.121	0.348	0.123	0.291	8	7
R2L-R3L	-0.529	-0.293	-0.057	-0.293	0.266	0.119	0.330	5	5
R2L-L3L	0.239	0.388	0.672	0.442	0.232	0.104	0.289	5	5
R1D-R3D	0.015	0.170	0.746	0.244	0.445	0.134	0.299	11	9
R1D-L3D	-0.044	0.197	0.495	0.200	0.387	0.103	0.223	14	11
R1D-R3L	-0.272	-0.028	0.421	0.054	0.350	0.157	0.435	5	5
R1D-L3L	-0.243	-0.033	0.175	0.035	0.394	0.149	0.364	7	7

Table S8. Synchronization index is the whole-chain preparation.

CS W-C	25%	Median	75%	Mean	SD	SE	±95% CI	n units	N animals
R1D-L1D	0.071	0.124	0.262	0.174	0.125	0.047	0.116	7	7
R2D-L2D	0.147	0.202	0.274	0.216	0.065	0.024	0.060	7	7
R2D-L2L	0.102	0.156	0.188	0.156	0.071	0.027	0.066	7	7
R2D-R2L	0.128	0.259	0.363	0.250	0.140	0.057	0.147	6	6
R3D-L3D	0.066	0.219	0.267	0.184	0.110	0.037	0.084	9	9
R3D-L3L	0.112	0.227	0.410	0.258	0.163	0.058	0.136	8	8
R3D-R3L	0.238	0.427	0.624	0.431	0.201	0.071	0.168	8	8
R1D-R2D	0.060	0.092	0.241	0.147	0.099	0.027	0.060	13	13
R1D-L2D	0.094	0.183	0.237	0.169	0.083	0.021	0.044	16	16
R1D-R2L	0.113	0.140	0.167	0.140	0.034	0.015	0.042	5	5
R1D-L2L	0.133	0.146	0.217	0.167	0.052	0.021	0.055	6	6
R2D-R3D	0.123	0.195	0.251	0.189	0.065	0.021	0.047	10	9
R2D-L3D	0.106	0.155	0.253	0.182	0.109	0.034	0.078	10	9
R2D-R3L	0.236	0.268	0.356	0.277	0.073	0.028	0.068	7	7
R2D-L3L	0.140	0.190	0.314	0.223	0.102	0.042	0.107	6	6
R2L-R3D	0.070	0.097	0.233	0.140	0.086	0.038	0.107	5	5
R2L-L3D	0.051	0.088	0.199	0.115	0.077	0.027	0.064	8	7
R2L-R3L	0.061	0.122	0.187	0.123	0.065	0.029	0.081	5	5
R2L-L3L	0.122	0.135	0.256	0.178	0.084	0.038	0.105	5	5
R1D-R3D	0.085	0.170	0.291	0.201	0.116	0.035	0.078	11	9
R1D-L3D	0.083	0.144	0.204	0.152	0.086	0.023	0.050	14	11
R1D-R3L	0.089	0.098	0.256	0.157	0.103	0.046	0.128	5	5
R1D-L3L	0.080	0.115	0.152	0.134	0.108	0.041	0.100	7	7

Table S9. Coupling strength is the whole-chain preparation.
